# Evidence of a fixed internal gene constellation in influenza A viruses isolated from wild birds in Argentina (2006–2016)

**DOI:** 10.1038/s41426-018-0190-2

**Published:** 2018-11-28

**Authors:** Agustina Rimondi, Ana S. Gonzalez-Reiche, Valeria S. Olivera, Julieta Decarre, Gabriel J. Castresana, Marcelo Romano, Martha I. Nelson, Harm van Bakel, Ariel J. Pereda, Lucas Ferreri, Ginger Geiger, Daniel R. Perez

**Affiliations:** 1 Instituto de Virologia CICVyA – Instituto Nacional de Tecnología Agropecuaria (INTA), CC25 (1712) Castelar, Buenos Aires Argentina; 2 Poultry Diagnostic and Research Center, College of Veterinary Medicine, University of Georgia, 953 College Station Rd, Athens, GA 30602 USA; 3 Instituto de Recursos Biológicos CIRN – Instituto Nacional de Tecnología Agropecuaria (INTA), CC25 (1712) Castelar, Buenos Aires Argentina; 4 Dirección de Áreas Naturales Protegidas, Organismo Provincial para el Desarrollo Sostenible (O.P.D.S), Gobierno de la provincia de Buenos Aires, General Conesa, Buenos Aires Argentina; 5 Centro de Investigaciones en Biodiversidad y Ambiente, Rosario (ECOSUR), Rosario, Santa Fe Argentina; 6 Fogarty International Center, National Institutes of Health, Bethesda, MD 20894 USA; 7 Department of Genetics and Genomic Sciences, Icahn School of Medicine at Mount Sinai, New York, NY 10029 USA; 8 Present Address: Instituto de Patobiología CICVyA – Instituto Nacional de Tecnología Agropecuaria (INTA), CC25 (1712) Castelar, Buenos Aires Argentina

## Abstract

Wild aquatic birds are the major reservoir of influenza A virus. Cloacal swabs and feces samples (*n* = 6595) were collected from 62 bird species in Argentina from 2006 to 2016 and screened for influenza A virus. Full genome sequencing of 15 influenza isolates from 6 waterfowl species revealed subtypes combinations that were previously described in South America (H1N1, H4N2, H4N6 (*n* = 3), H5N3, H6N2 (*n* = 4), and H10N7 (*n* = 2)), and new ones not previously identified in the region (H4N8, H7N7 and H7N9). Notably, the internal gene segments of all 15 Argentine isolates belonged to the South American lineage, showing a divergent evolution of these viruses in the Southern Hemisphere. Time-scaled phylogenies indicated that South American gene segments diverged between ~ 30 and ~ 140 years ago from the most closely related influenza lineages, which include the avian North American (PB1, HA, NA, MP, and NS-B) and Eurasian lineage (PB2), and the equine H3N8 lineage (PA, NP, and NS-A). Phylogenetic analyses of the hemagglutinin and neuraminidase gene segments of the H4, H6, and N8 subtypes revealed recent introductions and reassortment between viruses from the Northern and Southern Hemispheres in the Americas. Remarkably and despite evidence of recent hemagglutinin and neuraminidase subtype introductions, the phylogenetic composition of internal gene constellation of these influenza A viruses has remained unchanged. Considering the extended time and the number of sampled species of the current study, and the paucity of previously available data, our results contribute to a better understanding of the ecology and evolution of influenza virus in South America.

## Introduction

Influenza A Viruses (IAVs) belong to the family *Orthomyxoviridae*. The genome of IAVs is composed of 8 segments of negative-sense, single-stranded RNA. Wild aquatic birds of the order Anseriformes and Charadriiformes are considered the natural hosts of IAVs. Based on the antigenic characteristics of their surface glycoproteins, hemagglutinin (HA) and neuraminidase (NA), IAVs are divided into subtypes. To date, 16 HA subtypes (H1 to H16) and 9 NA subtypes (N1 to N9) have been recognized in wild birds. Due to the segmented nature of their genomes, IAVs frequently exchange gene segments, which has led to the identification of many subtype combinations in waterfowl species^1^. Regardless of the subtype, IAV infections in wild aquatic birds occur mostly in the intestinal tract in an apparently asymptomatic fashion. The virus is excreted in large amounts in the feces, which allows for an efficient fecal-oral route mode of transmission among birds.

Many of the efforts to understand the ecology of IAVs in their natural reservoir have been driven by a desire to track the origin of pandemic strains; prompted in particular by realization that the pandemic IAV viruses of the 1957 Asian flu (H2N2) and of the 1968 Hong Kong flu (H3N2) carried gene segments (HA, NA, and PB1; and HA and PB1, respectively) similar to IAV strains from ducks^2^. Renewed interest in understanding the ecology of IAVs in natural hosts resulted from the emergence of the HPAIVs of the H5 subtype in China with the ability to cause exacerbated and often lethal respiratory disease in humans. The HA gene segment of these viruses has continued to evolve and diversify into genetically distinct clades and sub-clades and have successfully spread across 4 different continents^3,4^. Historically, long-term active IAV surveillance in wild birds has been circumscribed to the United States, Canada, Western Europe, Southeast Asia, and China. In other parts of the world, surveillance efforts have been mostly passive, in response to disease outbreaks^5,6^. As a result, only 0.1% of all available sequences from IAV isolated from wild birds originate from South America^7^. The first evidence of IAV in birds in South America was associated to an outbreak of highly pathogenic avian influenza virus (HPAIV) of the H7 subtype in commercial chickens and turkeys in Chile in 2002^8^.

The evolution of IAVs in the natural reservoir is largely determined by the geographical separation and migratory patterns of the avian hosts. Thus, IAVs are broadly separated into lineages that correspond to vast geographic regions. IAVs of North American (NAm), Eurasian (EAs), and Australian lineages are well defined due to the significant amount of sequence information of IAV isolates available from those regions^9–11^. More recently, additional lineages were proposed for South America^12–14^ and Antarctica^7^ based on phylogenetic analyses of a limited number of IAV isolates obtained from wild birds.

Our group has been conducting active IAV surveillance in wild birds in Guatemala (Central America) and in Argentina (South America) as part of efforts to establish early warning systems for the potential introduction of IAVs from Asia into the Americas and/or from wild birds into commercial poultry. In the present study, we performed full genomic characterization and phylogenetic analyses of 15 low pathogenic avian influenza viruses (LPAIVs) isolated from 6 different avian species in Argentina between 2006 and 2016. We inferred time-scaled phylogenies using a previously described host specific local clock model^15^ using currently available sequences of IAVs from South America. Our studies provide further support for the existence of a South American (SAm) IAV lineage circulating in wild birds in Argentina. Interestingly, while most HA and NA subtypes obtained from the IAVs from Argentina follow unique evolutionary pathways within the SAm lineage, the HA and NA gene segments of three subtypes, H4, H6, and N8, appear to be recent introductions from IAVs of the NAm lineage. These analyses provide novel insights into the evolutionary patterns of these viruses. Taken together, our study highlights the importance of continuous surveillance of IAVs in South America as an integral part of worldwide efforts to better understand the ecology of these viruses and to better monitor the circulation of strains with zoonotic and/or pandemic concern.

## Results

### IAVs isolated from wild birds in Argentina with subtype combinations and from hosts not previously described in the region

**Fig. 1:**
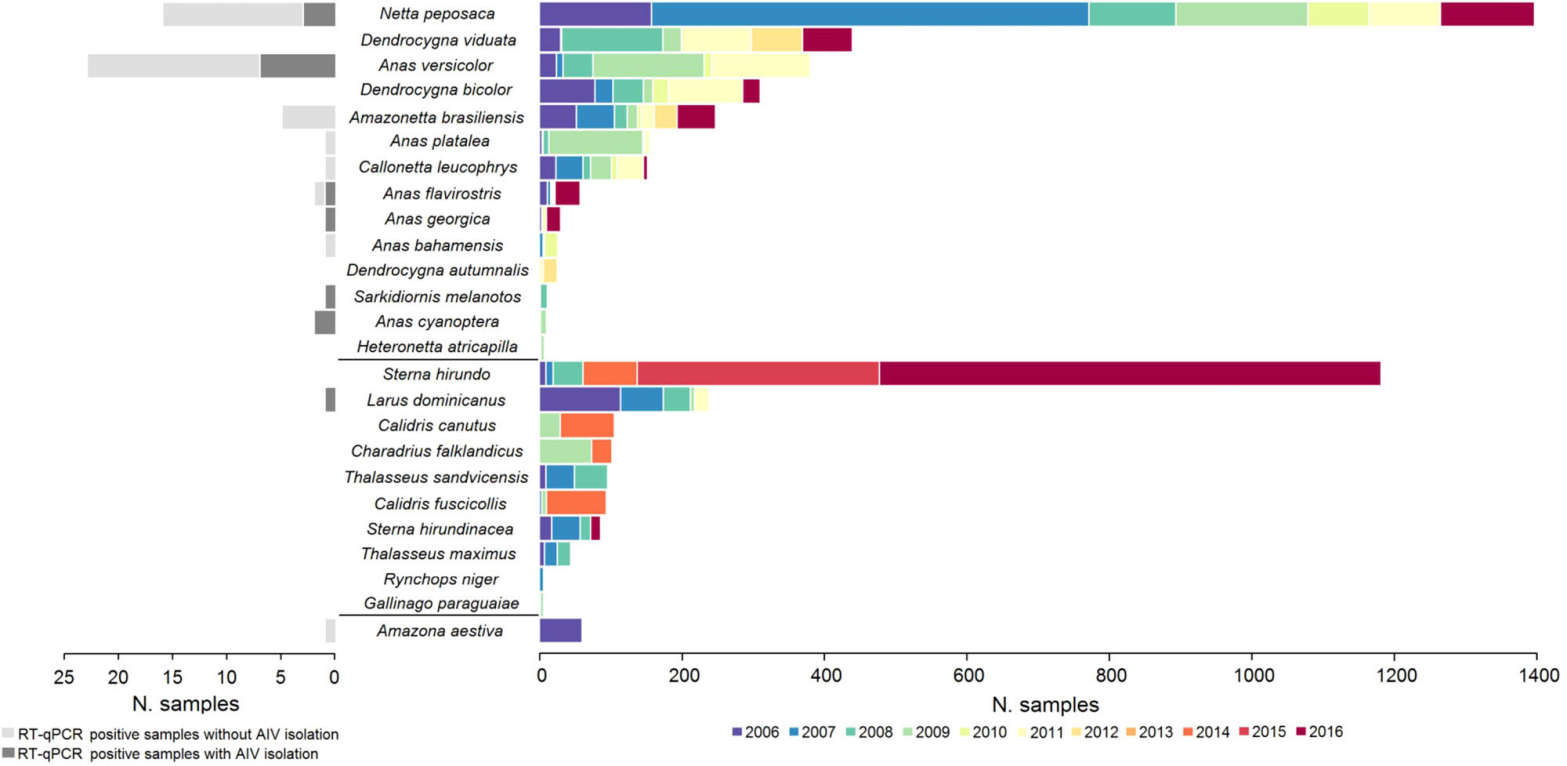
Avian species sampled for IAV detection from 2006 to 2016 in Argentina. Species belong to orders Anseriformes (upper 14 species), Charadriiformes (middle 10 species) and Pscittaciformes (bottom species). Right panel: only species with more than 5 samples collected are shown. Left panel: number of isolates per species obtained from RT-qPCR positive samples

In total, 6525 cloacal swabs and 70 fecal samples representing 62 different bird species were collected and tested for the presence of IAV (Fig. 1 and STable 1). Birds of the order Anseriformes (*n* = 3258; 49.4%), Charadriiformes (*n* = 1973; 29.9%) and Sphenisciformes (*n* = 744; 11.3%) comprised the majority of the birds sampled. Of these samples, 55 tested positive for IAV by RT-qPCR (0.83%), in their majority from ducks (*n* = 53), one from gull (*Larus dominicanus*) and one from parrot (*Amazona aestiva)* (STable 2).

**Fig. 2:**
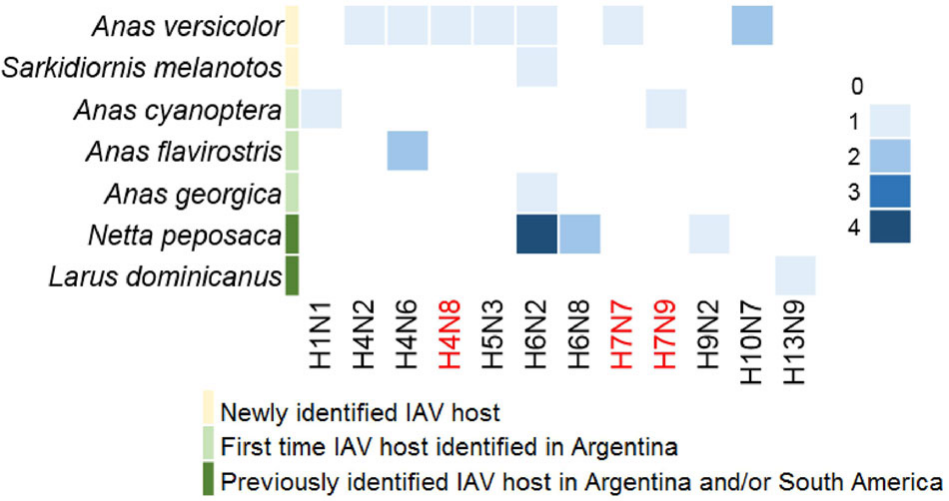
Avian-origin influenza virus subtypes obtained from avian species in Argentina (2006–2016). The gradient in color intensity indicates the number of isolates obtained for each subtype combination. Novel IAV subtypes, reported for the first time in South America, are shown in red

Viable IAVs were isolated from twenty-two RT-qPCR positive samples representing seven different bird species (Fig. 2), including *Anas versicolor* (silver teal, *n* = 8), *Netta peposaca* (rosy-billed pochard, *n* = 7), *Anas cyanoptera* (cinnamon teal *n* = 2), *Anas flavirostris* (yellow-billed teal, *n* = 2), *Anas georgica* (yellow-billed pintail, *n* = 1), *Sarkidiornis melanotos* (comb duck, *n* = 1) and *Larus dominicanus* (kelp gull, *n* = 1). Of these isolates, 7 have been previously described (subtypes H6N2 (3), H6N8 (2), H9N2 and H13N9, STable 3)^12–14^. Full genome sequences of the additional 15 IAV isolates (HA subtypes H1, H4, H5, H6, H7, and H10, and NA subtypes N1, N2, N3, N6, N7, N8, and N9) were generated for further characterization (STable 3). The subtype combinations H4N8, H7N7 and H7N9 have not been reported previously in South America (shown in red in Fig. 2). Previously unknown IAV hosts were also identified including *Anas versicolor* (silver teal) and *Sarkidiornis melanotos* (comb duck). Isolates from *Anas cyanoptera* (cinnamon teal), *Anas flavirostris* (yellow-billed teal), and *Anas georgica* (yellow-billed pintail) were obtained for the first time in Argentina (Fig. 2).

**Fig. 3:**
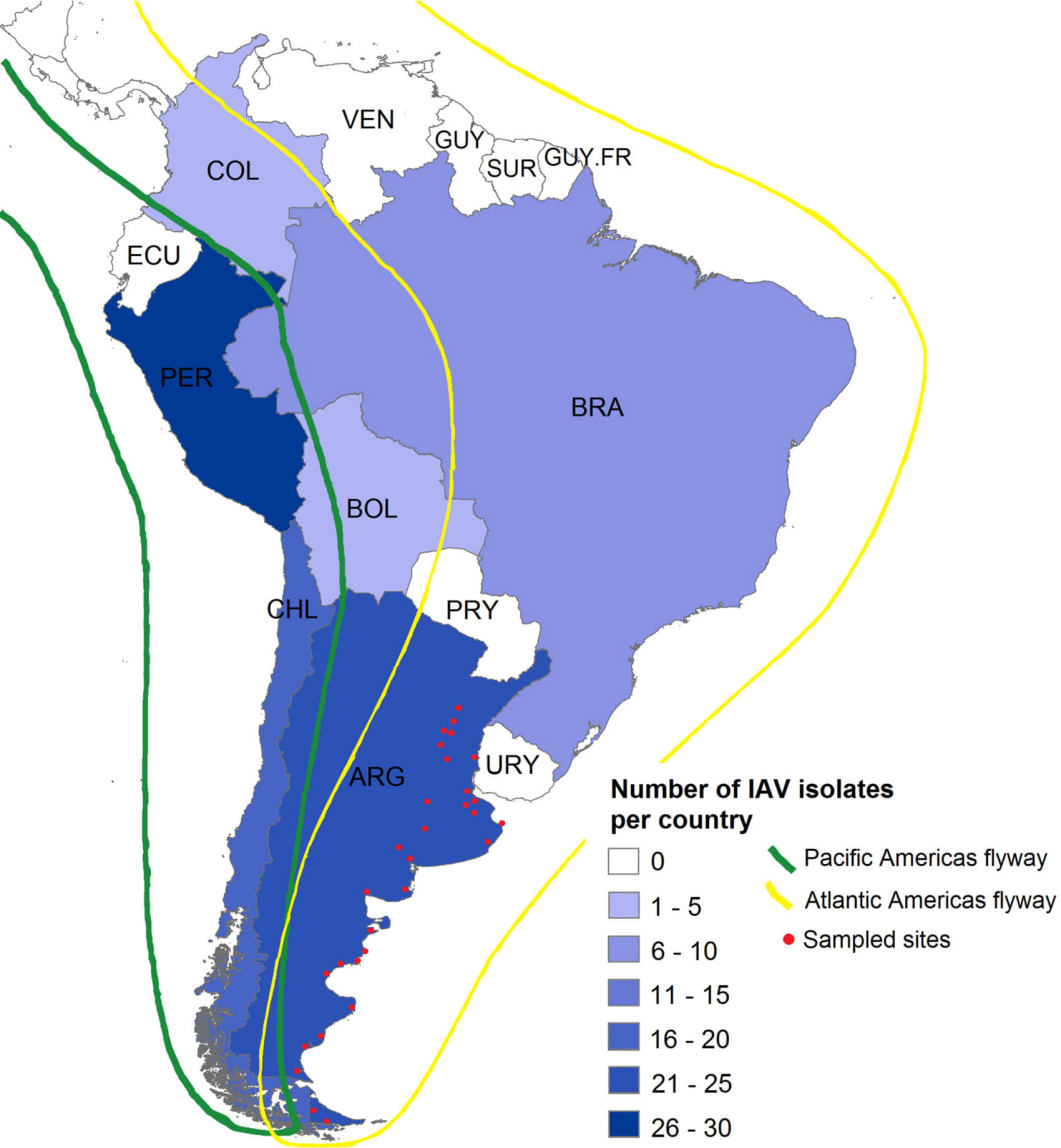
Number of IAV isolates in South America. The color intensity gradient indicates the number of IAVs obtained from wild birds in each country. Argentina (*n* = 23), Bolivia (*n* = 1), Brazil (*n* = 10), Chile (*n* = 20), Colombia (*n* = 2), and Peru (*n* = 30). Bird migration routes are indicated in green and yellow. Red dots represent all sites sampled for IAV in Argentina

Together, the fifteen Argentinean IAVs account for nearly seventeen percent of IAVs obtained from wild birds throughout South America. With these data, Argentina is the country with the second-highest number of IAV isolates in the Southern Hemisphere (Fig. 3).

### Phylogenetic analyses of internal gene segment constellations

**Fig. 4:**
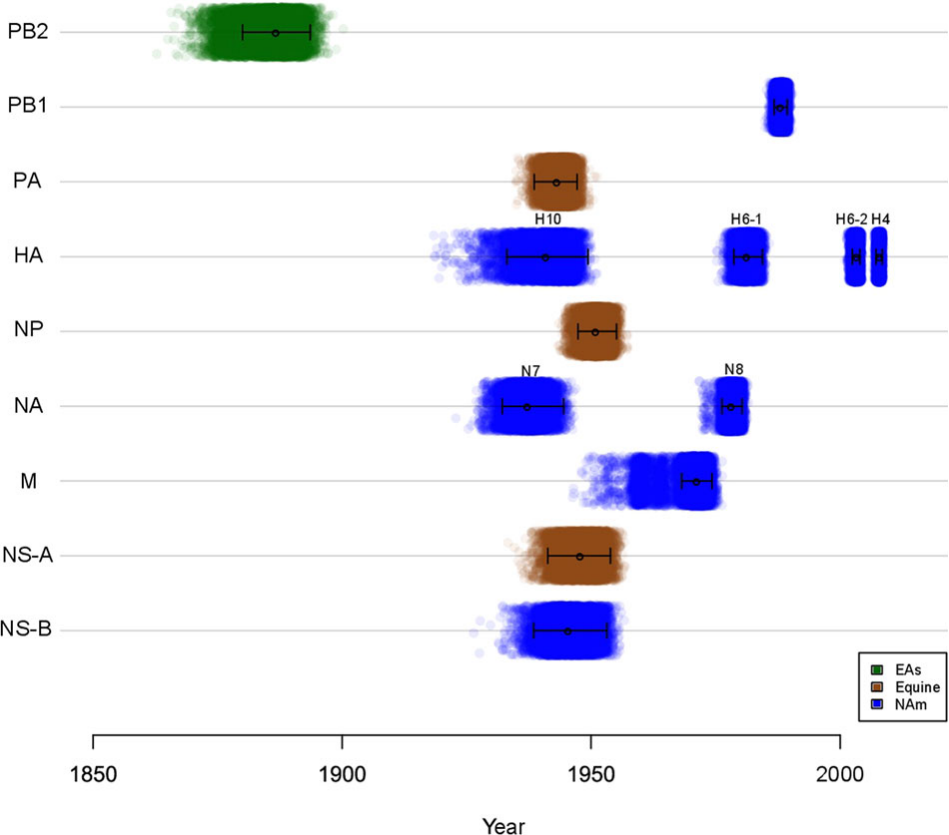
Time of divergence of the South American (SAm) lineage. Each dot represents the time of the most recent common ancestor, or node, between the SAm lineage and the most closely related lineage of each of 10,000 trees inferred for each segment. The identity of the most closely related lineage is shown by the colors. Mean tMRCA and 95% HPDs are represented by whiskers. Eurasian (EAs), North American (NAm)

The major open reading frame (ORF) sequence of each internal gene segment was used for phylogenetic analyses. Given prior evidence of possible common ancestry, sequences derived from equine IAVs were included in our analyses^13,16,17^. Prototypic nucleotide IAV sequences from other hosts (bats, swine, and human—except 2009 pandemic H1N1) were also included. The internal gene segments of the new 15 IAVs analyzed clustered together and are highly similar to other previously reported IAVs of avian origin from South America (SFig. 1–6). We used molecular clock analyses that incorporate host-specific evolutionary rates of IAV gene segments^15^ to estimate the time of the most recent common ancestor (tMRCA) for the internal gene segments of the IAV isolates from Argentina (Fig. 4 and STable 4), which indicated that these viruses diverged from other main IAV lineages between ~ 30 and ~ 140 years ago. A closer examination of each tree shows different topologies for each gene segment that indicate that the modern SAm lineage arose from multiple reassortment events, the exact timing of which remain unclear. The PB2 segment of the IAV strains from Argentina was introduced from an EAs-like IAV ancestor (Fig. 4 and SFig. 1) in the late 1880’s (tMRCA_PB2_ ~ 1887), in agreement with the previous analyses^15^. For this segment, the SAm avian clade diverged from the EAs lineage sometime between 1885 (tMRCA between EAs and SAm clades) and 1990 (tMRCA for SAm clade), implying a ~ 100-year gap of unknown diversity for the ancestors of the contemporary SAm clade. In contrast, the PB1 segment (Fig. 4 and SFig. 2) diverged more recently from a NAm lineage-like ancestor (tMRCA_PB1_ ~ 1988). Interestingly, the PA (tMRCA_PA_ ~ 1943, Fig. 4 and SFig. 3) and NP (tMRCA_NP_ ~ 1951, Fig. 4 and SFig. 4) segments diverged from the West equine-like (H3N8) lineage (Fig. 4 and STable 4) and share common ancestry with other IAVs from South America, Antarctica, and other equine-origin IAVs^7,18^. Similar to the PB1, the M segment diverged more recently from a NAm lineage ancestor (tMRCA_M_ ~ 1971, Fig. 4 and SFig. 5) (STable 4).

Phylogenetic analysis of the NS genes showed that 13 of the IAVs from Argentina contain the NS allele A (NS-A), whereas only two carry allele B (NS-B) (SFig. 6). Both of these alleles diverged from the main IAV lineages around the same time, between 1939 and 1954 (Fig. 4 and STable 4). Like the PA and NP segments, the NS-A gene segments form a sister group with IAVs of equine origin^13,16,17^.

**Fig. 5:**
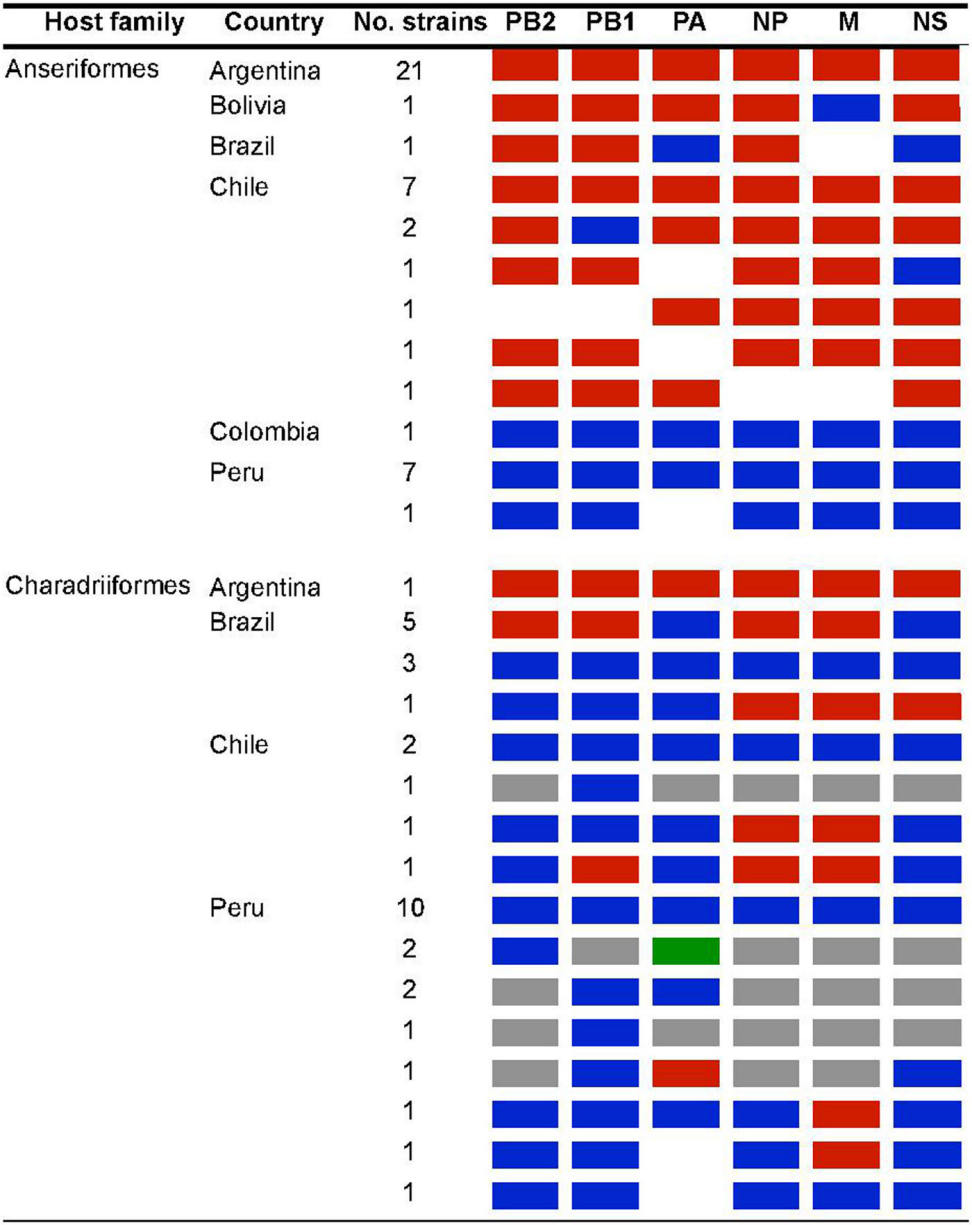
Genomic constellations of avian-origin IAVs from South America. IAVs by host-order and country color-coded as South American lineage (red), North America lineage (blue), Eurasian lineage (green), other global avian lineages (gray), and no sequence data available (white)

Analysis of the internal gene segment constellations for all Argentinean LPAIV strains and other viruses obtained from wild aquatic birds and poultry in South America revealed that all viruses from Argentina are positioned exclusively within the South American lineage. In contrast, viruses from Bolivia, Brazil, Chile, Colombia, and Peru include reassortants with IAVs of other genetic lineages (Fig. 5, STable 5 and SFigs. 1–6).

### Phylogenetic analyses of the surface gene segments

**Fig. 6:**
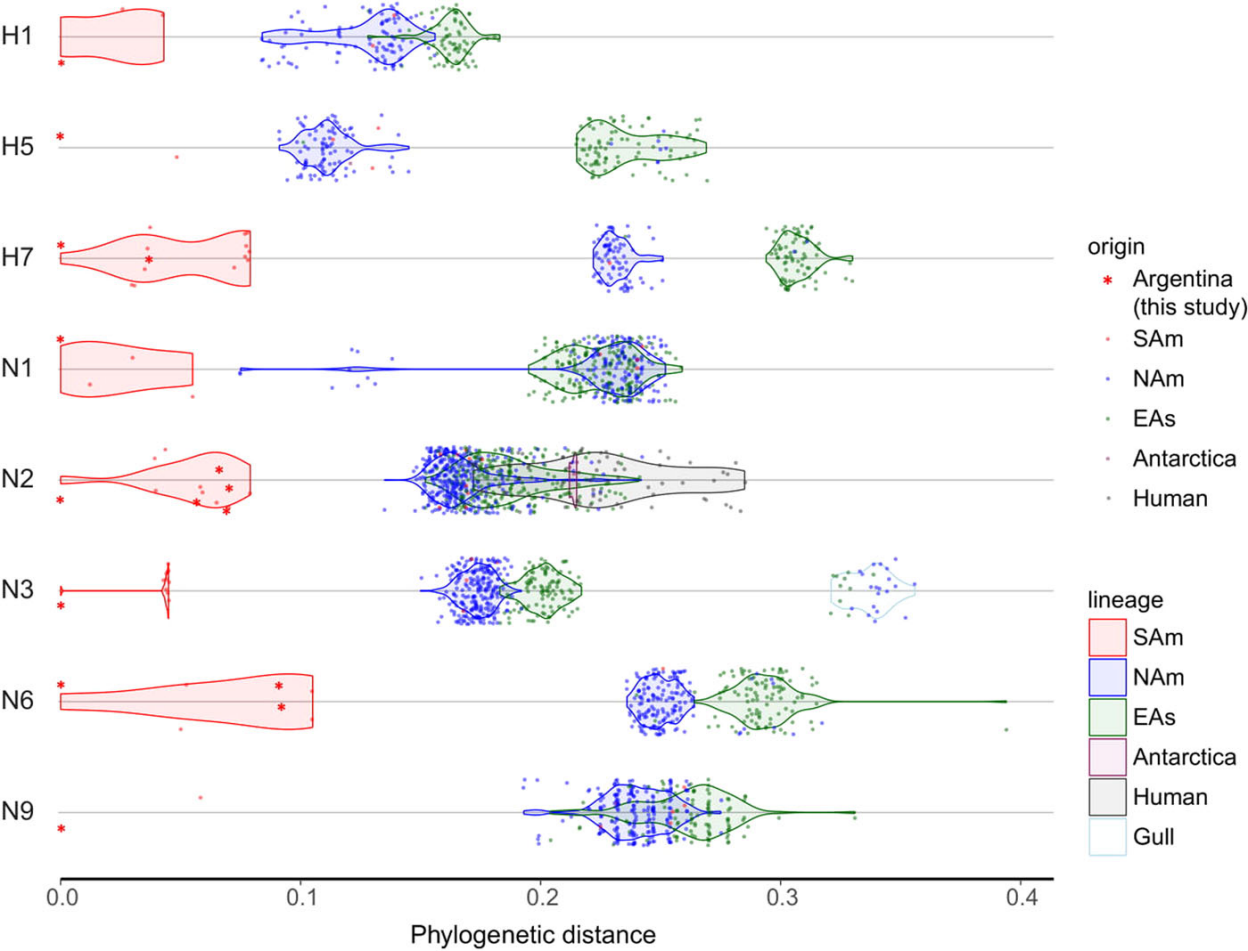
Phylogenetic distance (number of base substitutions per site) between a reference sequence from an IAV from Argentina and other global LPAIV sequences used for phylogenetic analysis. Each dot represents a single sequence (virus) and it is colored by its source (geographic or host). Argentinean IAVs are shown with red asterisks. The continuous distribution of viruses within their main lineages is shown by the violin shapes for lineages with more than 2 observations: avian South American (SAm), North America (NAm), Eurasian (EAs). Argentinean isolates used as references and shown at zero distance: 432/H1N1 (H1 and N1), 1737/H5N3 (H5 and N3), 188/H7N7 (H7), 1174A/H6N2 (N2), 1227/H4N6 (N6), 1588/H7N9 (N9). Detailed phylogenies are provided in the supplementary material

Overall the HA gene segments of Argentinean IAVs clustered with other South American IAVs (H1, H5, H7, H4, and a subset of H6, named H6-2), with the exception of the H10 and a subset of H6 (H6-1) gene segments that formed a cluster of IAVs exclusively from Argentina. We denoted all these clades as SAm lineage. Following is a description of the main findings for each HA subtype. The H1 and H5 isolates from Argentina grouped within the NAm lineage (Fig. 6 and SFigs. 7 and 8). The H1 subtype SAm lineage viruses were recovered from resident wild birds (Anseriformes and Tinaniformes) from Argentina and Chile (Fig. 6 and SFig. 7). The other H1 subtype IAVs identified in Peru (Charadriiformes and Gruiformes) belongs to the NAm lineage (Fig. 6 and SFig. 7). The H5 subtype SAm lineage contains only two sequences from IAVs from resident duck species (silver teal and yellow-billed pintail) in Argentina and Chile. There were other two highly similar H5 sequences in the database (from Chile), but they were excluded from the analysis because they were only partial fragments of the H5. In addition, there are other H5 subtype IAVs isolated from different wild duck spp. in South America (Chile and Colombia), but they cluster in a different position within the NAm lineage and are less divergent compared to the Argentinean isolate (Fig. 6 and SFig. 8). The H7 subtype from IAVs from the SAm lineage was basal to the main NAm lineage, showing two clades (SFig. 9). One clade contains the two H7 IAVs from Argentina and other IAVs from Chile (all IAV isolates from resident wild duck spp.). The second clade contains the HPAIV poultry isolates from the outbreak in Chile in 2002 and their closest wild birds LPAIV ancestor from Bolivia (cinnamon teal) (refs. ^8,17^). Although all H7 from Argentina are LPAIV, they share a common ancestor with H7 HPAIVs from 2002. Similar to the H5 subtype, the other H7 subtype identified from a wild duck (unknown spp.) in Peru belongs to the NAm lineage (Fig. 6 and SFig. 9).

The H10 subtype has been previously reported in Anseriformes and Charadriiformes in Peru^19^. For this subtype, the two H10N7 IAV isolates from Argentina grouped together and formed a unique branch that is clearly separated from H10 gene segments from viruses from other geographic locations, and remarkably distant from the H10N7 viruses from Peru (SFig. 10, red branches). As this was the first isolation of a SAm H10 subtype, we inferred an MCC tree to estimate the time of divergence of these viruses. Both H10 Argentinean viruses, isolated from silver teal, have a common ancestor with H10 viruses from NAm lineage, with an estimated tMRCA between 1933 and 1949 (95% HPD) (Fig. 4 and STable 4).

Phylogenetic analysis of H4 HA gene segment showed that the IAVs from Argentina (four isolates from 2011 and one from 2016) grouped within the NAm lineage and were closely related to other H4 viruses identified in Anseriformes in Chile in 2013 and 2015, suggesting dissemination of NAm H4 subtype IAVs in the region (SFig. 11). As this seemed to be a recent introduction to South America we estimated the time of introduction for this clade. Time-scaled phylogenetic analysis estimated that the introduction of these NAm H4 viruses in South America occurred between 2007–2009 (95% HPD) (Fig. 4 and STable 4). Similarly, the MCC tree of H6 HA gene showed at least two separate introductions of H6 viruses in Argentina (identified as H6-1 and H6-2 in Fig. 4 and SFig. 12). The first introduction (H6-1) includes two new H6 isolates (272/H6N2 and 1174A/H6N2) that clustered together with four H6 IAVs from Argentina published previously^13^. In this case, we estimated that the SAm clade (as well as the most closely related NAm clade) has diverged from the EAs lineage sometime between 1978 and 1984 (95% HPD, Fig. 4 and STable 4). The second most recent introduction (H6-2) includes other two new H6 isolates (49/H6N2 and 52/H6N2) that grouped together with one of our previous H6 subtype IAVs (1977/H6N2) and three IAVs from Brazil isolated in 2012 (SFig. 12). On this occasion, the SAm clade seems to have diverged from the NAm lineage sometime between 2002 and 2004 (95% HPD, Fig. 4 and STable 4). The new Argentinean H6 IAVs were obtained from four wild duck spp. (comb duck, rosy-billed pochard, silver teal, and yellow-billed pintail), while Brazilian H6 IAVs were obtained from a small shorebird (white-rumped sandpiper, a Charadriiforme).

For the NA gene segments, phylogenetic analysis of the N1, N2, N3, N6, and N9 gene segments grouped them into the unique SAm clades as previously observed^12–14^ (Fig. 6 and SFigs. 13–17). The N1 subtype SAm lineage contains four similar sequences, three are paired with the H1 HA subtype from IAVs isolated in Argentina and Chile (from Tinaniformes and Anseriformes respectively), and one is paired with the H6 HA subtype from IAVs isolated from white-rumped sandpiper in Brazil (SFig. 13). These four viruses form a sister group with some IAVs isolated from shorebirds in Delaware Bay and New Jersey in a cluster that is basal to the NAm and EAs lineages. The N1 of other IAVs identified from Chardriiformes in Brazil and Peru belong to the NAm lineage. The N2 phylogenetic tree shows IAVs from SAm lineages share ancestry with avian IAVs from diverse origins that are basal to the main NAm and EAs lineages. The SAm viruses grouped in two clades (SFig. 14), one clade contains IAVs isolated exclusively from Anseriformes in Argentina and Chile between 2007 and 2014 (*n* = 9, four new sequences from this report). The second clade contains one N2 subtype from an IAV isolated in Argentina in 2016 (this report) and N2 sequences from IAVs from Chile (2015). The other N2 IAVs identified in Chile, Colombia, and Peru belong to the NAm lineage. The N3 subtype from IAVs from the SAm lineage also shows two clades (SFig. 15). One clade contains only two H5N3 IAVs isolated from wild ducks, one from Argentina (this report) and the other from Chile. There is a 6-year gap between these IAVs (2009 and 2015) and they were isolated from different species—silver teal and yellow-billed pintail, respectively. The second clade contains only H7N3 HPAIV isolate from poultry in Chile in 2002 (ref. ^8^) (SFig. 15). These two clades shared ancestry with non-contemporary NAm viruses, that are basal to NAm lineage. The N6 subtype SAm lineage, basal to the NAm lineage, contains the three newly identified N6 IAVs from Argentina and other sequences from Chile, all from resident wild bird spp. (SFig. 16). The only other N6 IAV identified in South America, in Peru, belongs to the NAm lineage and was isolated from a gull. Interestingly, our last N2 and N6 IAV isolates from 2016 (1174A/H6N2 and 1227/H4N6 respectively) are more distant from previous isolates from Argentina and more closely related to recent N2 and N6 segments from IAVs isolated in Chile (SFig. 14 and 16 respectively). With respect to the N9 gene segments, those obtained from IAVs in Argentina (*n* = 2) were basal to the main EAs and NAm lineages, whereas N9 subtype sequences from other IAV isolates from Brazil, Chile and Peru clearly belong to the NAm lineage (SFig. 17).

The N7 subtype phylogeny shows that sequences of those IAVs isolated in Argentina, all from silver teals, are unique and share no relationship with those obtained from wild bird isolates in Chile^20^ or Peru^19^ (SFig. 18, red branches). We estimated the time of divergence of these distinct viruses. The N7 subtype sequences from IAVs from Argentina have a NAm ancestor with a tMRCA between 1932–1945 (95% HPD), similar to the H10 HA subtype sequences (Fig. 4 and STable 4).

Finally, three N8 IAVs from Argentina clustered together as a distinct clade within the NAm lineage. Time-scaled phylogenetic analysis estimated that the time of introduction of the N8 subtype in Argentina was between 1976–1980 (95% HPD) (Fig. 4 and STable 4), which overlaps with the first introduction of H6 HA in SAm, suggesting common origins in a NAm ancestor. The N8 NA phylogenetic tree revealed that previously described sequences from IAVs from wild birds (not Anseriformes) in Peru^19^ also belong to the NAm lineage but represent a more recent, independent introduction, different from Argentina (red branches SFig. 19).

### Molecular analyses of virulence markers

The diversity of HA subtypes found in IAVs from Argentina confirm the circulation of group 1 (H1, H5, H6, H9, and H13) and group 2 (H4, H7, and H10) of HAs previously proposed^21^. Previous studies have demonstrated the amino acid changes in the receptor binding site at positions 190, 225, 226, and 228 of the HA protein (H3 numbering) in a variety of subtype, including H1, H2, H3, H4, H5, H6, and H7^22–25^. Analysis of predicted HA amino acid sequences showed that all Argentinean viruses isolated from 2006 to 2016, regardless of subtype, contain amino acid residues consistent with binding to “avian-like” α2,3 sialic acid receptors (E190, G225, Q226, and G228, data not shown). In addition, 16 out of 22 Argentinean IAV strains shared potential glycosylation sites of asparagine (Asn) along the HA1 domain (H3 numbering) with IAVs previously isolated in other regions: Asn129^26^ for 432/H1N1 strain; Asn6, Asn22, Asn165, Asn196, and Asn483^27^ for all H4 strains (*n* = 5); Asn158^28^ for 1737/H5N3 strain; and Asn21, Asn33, Asn170, Asn291, and Asn296^29^ for all H6 strains (*n* = 9). Instead, the 188/H7N7 and 1588/H7N9 strains maintain Asn133 but have the mutation N158T that abolishes a potential glycosylation site.

The HA gene segments of the H1, H4 (*n* = 5), H5, H6 (*n* = 9), H9 and H13 subtypes, have cleavage site motifs typical of LPAIV (Table 1). The HA gene segment of the H7 (*n* = 2) and H10 (*n* = 2) subtypes, have cleavage sites different from the consensus sequences described by Baron et al.^30^ (Table 1). In addition to the arginine at the P1 position, the H7 HA cleavage site contains basic amino acids (lysine) at the P3 and P5 positions, and the H10 HA cleavage site carries histidine at the P3 position. It remains to be determined if such cleavage site motifs modulate the virulence of these viruses in wild birds and/or poultry.

**Table 1:** Cleavage site motifs of avian-origin influenza A viruses isolated in Argentina

Virus	Amino acid consensus sequence at the HA cleavage site (subtype)^a^	HA cleavage site^b^	Amino acid position in HA0	Virulence^c^
432/H1N1^d^	PSIQS**R**G (H1)	PSIQA**R**G	339–345	Avirulent
32/H4N2^d^	PEKAS/T**R**G (H4)	PEKAT**R**G	338–344	Avirulent
48/H4N6^d^		PEKAT**R**G	338–344	Avirulent
91/H4N6^d^		PEKAT**R**G	338–344	Avirulent
1227/H4N6a		PEKAT**R**G	338–345	Avirulent
25/H4N8^d^		PEKAT**R**G	338-344	Avirulent
1737/H5N3^d^	PQRET**R**G (H5)	PQRET**R**G	337–343	Avirulent
272/H6N2^d^	PQIET**R**G (H6)	PQIET**R**G	339–345	Avirulent
557/H6N2		PQIET**R**G	339–345	Avirulent
925/H6N2		PQIET**R**G	339–345	Avirulent
1977/H6N2		PQIET**R**G	339–345	Avirulent
49/H6N2^d^		PQIET**R**G	339–345	Avirulent
52/H6N2^d^		PQIET**R**G	339–345	Avirulent
1174 A/H6N2^d^		PQIET**R**G	339–345	Avirulent
269/H6N8		PQIET**R**G	339–345	Avirulent
575/H6N8		PQIET**R**G	339–345	Avirulent
188/H7N7^d^	PEI/NPKG/T**R**G (H7)	PEKPKT**R**G	333–340	Unkown
1588/H7N9^d^		PEKPKT**R**G	333–340	Unkown
559/H9N2	PAR/K/XS/LX/S**R**G (H7)	PAASN**R**G	333–339	Avirulent
171/H10N7^d^	PEI/VM/VQG/E**R**G (H10)	PEIIHE**R**G	334–341	Unkown
175/H10N7^d^		PEIIHE**R**G	334–341	Unkown
LDC4/H13N9	PAISN/T**R**G (H13)	PAISN**R**G	344–350	Avirulent

^a^From Table 2 in Baron J et al., J Virol. 2013 Feb; 87(3): 1811–1820. The basic amino acid at the cleavage site is shown in bold

^b^The basic amino acid at the cleavage site is shown in bold. Amino acid at the HA cleavage site different from amino acid consensus sequences are underlined

^c^Virulence phenotype inferred from protein sequence analysis

^d^New fifteen IAVs isolates from Argentina from this study

No mammalian-associated virulence markers in NS1 protein (T92E)^31^ or PB2 (E627K and D701N)^32–34^ were found in the viruses from Argentina (STable 6). None of the viruses contained mutations associated with drug resistance in the NA and M2 proteins^35,36^, except for the 432/H1N1 virus, which carries the mutation NA H274Y predictive of oseltamivir resistance^37^. No mutations associated with enhanced transmission in mammals were found in the PB2 and PA proteins^34,38^. In comparison to other lineages (NAm and EAs), none of the amino acid residues associated with drug resistance and virulence described here were unique to the Argentinean viruses or the SAm clade (STable 6). High degree of sequence homology at the protein level of the SAm lineage with other lineages was previously observed^13^.

The IAVs from Argentina contain the typical PB1-F2 ORF of 90 amino acids in length commonly found in IAVs from ducks^39,40^. There are 45 amino acid differences among the PB1-F2 proteins in the IAVs from Argentina (SFig. 20). Interestingly, the PB1-F2 of Argentinean strains carry the N66S mutation implicated in increase virulence for mammals^41^, with the exception of the 52/H6N2 strain. In addition, the PB1-F2 protein of the 1227/H4N6 strain contains three amino acid markers, 51T, 56V, and 87E, associated with virulence in mallards of a highly pathogenic H5N1 avian influenza virus^42^. Other Argentinean strains contain the 56V and 87E markers, the 1977/H6N2 contains only the 87E and the 1174A/H6N2 and 559/H9N2 strains contain only the 56V marker. Since the effects of PB1-F2 are virus- and host-dependent, further studies are needed to better understand the role of these PB1-F2 variants in the IAVs from Argentina^40,41,43^.

The IAVs from Argentina also contain the predicted full-length PA-X ORF of 252 amino acids in length. There are 24 amino acid differences among the PA-X proteins in the IAVs from Argentina (SFig. 21). As is the case for PB1-F2, the effects of PA-X on virus replication and pathogenesis are strain- and host-dependent^44,45^, and further studies are necessary to determine the biological significance of these mutations in birds.

## Discussion

Long-term IAV surveillance in wild waterfowl in Argentina between 2006 and 2016 resulted in the recovery of 22 isolates, 15 of which are newly reported here. Despite limited and opportunistic sampling across host species and locations within Argentina throughout the 10 years of the study (shown as red dots in Fig. 3), we consistently observed that all internal segments of the Argentinean viruses were closely related to those from viruses previously isolated in Argentina, and exclusive to this geographical location in South America. Taken together, the results provide additional support for the existence of a unique SAm avian lineage with local independent evolution for all internal viral gene segments^12–14,17^.

The phylogenetic analysis of the internal gene segments from the Argentinean IAVs showed shared ancestry with equine-origin IAVs (PA, NP, NS-A), and avian-origin IAVs of Eurasia (PB2), North America (PB1, M, NS-B) and Antarctica (PA, M), demonstrating that their genetic diversity has been shaped by multiple introductions and reassortment occurring as early as the end of the 19th century^15^. Interestingly, the IAVs isolated from wild birds in Argentina have all internal gene segments of SAm origin, while many Chilean and Peruvian viruses have either all or most of their internal genes segments of NAm origin, and close to those from Central America^19,20,46^. More recently, H5N5 isolates from Antarctica were shown to carry gene constellations of NAm origin^7,18,47^. While reassortment of internal gene segments from IAV from different lineages do occur in parts of South America, the Argentinean IAVs circulate mostly in isolation with limited gene segment exchange with viruses from other latitudes, regardless of their subtype.

Phylogenies of the H1, H5, H7, and H10 HA subtypes and the N1, N2, N3, N6, N7, and N9 NA subtypes demonstrated that both genes follow unique evolutionary patterns in wild birds in Argentina. Although the primordial source of these viruses remains unknown, the results suggest that the Argentinean strains split from their ancestors, either Eurasian or North American, ≤ 30 years ago and continue an independent evolution pathway. It must be noted that our surveillance efforts in Argentina have covered limited areas of the country and a limited subset of avian species. Together with the scarce data available from IAVs in South America, it is difficult to confirm if the basal lineages of SAm viruses are ancestral to other main IAV lineages (for example NAm lineage) or whether they are a consequence of earlier virus introductions (from any lineage) that then diverged separately in this region. Expansion into broader geographic areas, animal species, and number of samples are indispensable as a first step to better understand the ecology and evolution of IAVs in wild birds in Argentina. Sequence analysis of the Argentinean IAVs are consistent with typical LPAIVs; however, there are remarkably divergent variants (e.g. H5, H7, H10) that warrant further experimental characterization in the laboratory to deepen our knowledge in the pathogenicity and host-range of these particular SAm IAVs.

In this study, we traced the origin of the Argentinean H4, H6, and N8 subtypes to North America, and detected at least four independent introduction events within the past 40 years. Particularly, the results of the H6 analyses further support the presence of two independent H6 subtype populations circulating in Argentina^13^. In addition, these two Argentinean H6 gene clusters are different from the H6 subtype population that circulates in Charadriiforms and Pelecaniformes in Peru, indicating distinct ways of dissemination of NAm H6 subtype IAVs along the region. The IAV gene influx from North America into South America could be driven by the migration of different bird species that arrive to the Southern Hemisphere through the Atlantic and Pacific flyways (Fig. 3). With the available data, however, the bird species involved in this process and the frequency of such events remains unclear.

At the level of internal gene constellations, the lack of reassortment between the viruses from Argentina and other latitudes is notable (Fig. 5 and STable 5), but may be the result of sampling bias in geographical locations and/or host species. Several IAVs from Chile and Peru were obtained from gulls and shorebirds that perform long migrations that serve as important circuits for the dissemination of IAV^48,49^. In contrast, Argentinean isolates were obtained almost exclusively from resident wild duck species with distribution more restricted to South America^50^. Systematic surveillance of a larger number of IAV isolates obtained from South America will be necessary to trace the true origin of the SAm lineage, and further understanding of population densities and regional movement among migratory and resident birds in Argentina and other SAm countries is crucial to understand the dynamics of IAV transmission. Monitoring the movement of wild birds by geolocators, and identifying species subpopulations through analysis of stable isotopes and molecular genotyping^51–53^, have the potential to establish the possible points of contact in which wild birds from North and South America overlap to allow virus transmission and reassortment. Such studies would also serve to elucidate whether geographic barriers, such as the Andes Mountains (natural border between Argentina and Chile), restrict the movement of wild bird populations and spillover of viruses from other genetic lineages (e.g., N.Am) into resident species, explaining the absence of reassortant virus strains in Argentina.

## Materials and methods

### Sample collection

Trained biologists, veterinarians, and park rangers performed sampling between April–July (waterfowl and resident birds) and October–March (shorebirds and migratory species), from 2006 to 2016. Samples were obtained from a variety of seabirds (gulls, cormorants, penguins, terns, and shorebirds) captured along the Argentine Atlantic coast, and hunter-killed ducks in the Lower Paraná River Valley, as described previously^12,13^.

Cloacal swabs were collected using single-use sterile polyester swabs and then stored separately in single plastic cryovials, containing 2 ml of phosphate buffer solution (PBS) with 50% glycerol and penicillin 10,000 IU/ml, streptomycin 5 mg/ml, gentamicin Sulfate 1 mg/ml, kanamycin sulfate 700 µg/ml and amphotericin B 10 µg/ml (Sigma Chemical Co, St. Louis, MO, USA). Samples were frozen in liquid nitrogen and transported on dry ice. Once in the laboratory, all samples were stored at −80 °C until processing for molecular diagnosis and virus isolation.

### Virus detection

Viral RNA was extracted from 140 µl of suspension from cloacal swabs using a QIAamp Viral RNA Mini Kit (Qiagen Inc., Valencia, CA, USA). RNA was eluted in a final volume of 60 µl and stored at −80 °C until further use. Viral cDNA was prepared using 15 µl of viral RNA and random hexamers in a final volume of 30 µl using a High Capacity cDNA Archive kit (Applied Biosystems, Foster City, CA, USA). The cDNA was tested for IAV by real-time reverse transcriptase PCR (RT-qPCR) using TaqMan Universal PCR Master Mix (Applied Biosystems) directed to the matrix (M) gene. Samples collected from 2006 to 2012 were tested using primers and probe previously described by Spackman et al.^54^, and samples collected since 2013 were tested using primers (InfA Forward and InfA Reverse) and probe (InfA Probe) described in WHO/CDC protocol titled “CDC protocol of real time RTPCR for swine influenza A(H1N1)” and “28 April 2009 revision 1 (30 April 2009).” The PCR reaction was performed on an ABI Prism 7500 SDS (Applied Biosystems). Both methods were validated for sensitivity against avian influenza viruses and no differences were obtained with respect to IAV detection (data not shown).

### Virus isolation

Swab samples that tested positive by RT-qPCR were inoculated into 9–11-day old specific-pathogen-free (SPF) embryonated chicken eggs. Briefly, 200 µl of PBS suspension from cloacal swab samples was injected into the allantoic cavity of the eggs, incubated for 72 h, and harvested in accordance with standardized protocols as suggested by the World Health Organization (http://www.wpro.who.int/emerging_diseases/documents/manual_on_animal_ai_diagnosis_and_surveillance/en/) and following Argentine regulations.

### Full genome sequencing and assembly by Next Generation Sequencing

PCR was performed using specific primers for each gene segment as described previously^55^. PCR products were purified with a QIAQuick PCR purification kit (Qiagen). Sequencing was performed using the BigDye Terminator v3.1 Cycle Sequencing Kit on an ABI PRISM 3700 DNA Analyzer (Applied Biosystems) following the manufacturer’s instructions. A subset of isolates (marked with “^#^” in STable 3) was re-sequenced by Next Generation Sequencing and nucleotide sequence identity from Sanger sequencing was verified. Briefly, products from a multisegment-RTPCR strategy^56^ were cleaned by 0.45X of Agencourt AMPure XP Magnetic Beads (Beckman Coulter) according to manufacturer’s protocol. Eluate concentration was measured using the Qubit High Sensitivity dsDNA kit (Fisher Scientific) in the Qubit 3.0 fluorometer (ThermoFisher) and normalized to 0.2 ng/ul. Adaptors were added by tagmentation using the Nextera XT DNA library preparation kit (Illumina). Samples were purified using 0.7X of Agencourt AMPure XP Magnetic Beads and fragment size distributions were analyzed on a Bioanalyzer using the High Sensitivity DNA kit (Agilent). Next, samples were normalized to 4 nM and pooled. The final loading concentration of the pooled libraries was 15pM. Libraries were sequenced in a paired-end run using the MiSeq v2, 300 cycle reagent kit (Illumina).

Genome assembly was performed using a customized pipeline developed at the Icahn School of Medicine at Mount Sinai^56^.

### Phylogenetic and molecular analysis

The consensus amino acid and nucleotide sequences for all 8 gene segments of the viruses were generated using Megalign (DNASTAR, Madison, WI, USA). Phylogenetic analyses were performed using additional influenza virus sequence data available in the Influenza Research Database (https://www.fludb.org/brc/home.spg?decorator = influenza), and were run with Maximum clade credibility in BEAST or Maximum Likelihood (ML) in MEGA (see below).

Time-scaled phylogenetic analyses were performed in the program BEAST v.1.8.2. For the virus internal gene segments, and surface glycoproteins N7 and N8, previously published datasets were downloaded from the Dryad repository^15^ with permission from the authors. Additional virus nucleotide sequences (2011–2016) were added to the original alignments, including all sequences from Argentina. Representative strains from South America were selected after inferring Maximum Likelihood phylogenetic trees for all internal gene segments available in IRD. The new alignments for PB2 (total number of sequences = 435), PB1 (*n* = 413), PA (*n* = 462), NP (*n* = 508), M (*n* = 519), NS (*n* = 442), N7 (*n* = 213) and N8 (*n* = 463) were performed with MUSCLE as implemented in the program MEGA 6.0. The MCC trees were inferred using a host-specific local clock model as previously described^15^, in which all host clades are permitted individual substitution rates. The parameters for the time-scaled phylogenetic analysis were set in the program Beauti, with manual edits for assignment of host-specific rates as specified in the original xml files^15^. We used the SRD06 model of nucleotide substitution^57^, and the coalescent tree prior GMRF Bayesian Skyride. For the H4, H6, and H10, the inferred NJ phylogenies were analyzed in the program Path-O-Gen to identify and remove outlier sequences. The final alignments of H4 (*n* = 264), H6 (*n* = 469) and H10 (*n* = 258) were used for phylogenetic analyses. The MCC trees were then inferred under the SRD06 model of nucleotide substitution, using the uncorrelated lognormal clock and a constant population size. For the mean rate, a prior of 0.001 was used in all cases. For each data set the MCMC chain was run for 20–100 million steps until convergence of parameters was observed in the program Tracer (v.1.6.0). For each chain, the first 10% states were removed (10% burn-in). Then the results of at least three independent runs were merged in the program LogCombiner. The MCC trees were summarized and annotated in TreeAnnotator, visualized and printed in FigTree (v.1.4.2). The times of divergence for the viruses from South America and Argentina were obtained from the node ages (and their corresponding 95% HPD) on the final MCC trees.

Maximum likelihood (ML) phylogenetic trees for H1 (*n* = 172), H5 (*n* = 183), H7 (*n* = 173), N1 (*n* = 339), N2 (*n* = 637), N3 (*n* = 380) and N6 (*n* = 264) and N9 (*n* = 297) gene segments were constructed by using MEGA 6.0 (https://www.megasoftware.net/), including the viruses from Argentina, other IAVs from South America and other background sequences representative of the LPAIV global diversity, available in Influenza Research Database (https://www.fludb.org/brc/home.spg?decorator=influenza). Estimates of the phylogenies were calculated by performing 1000 bootstrap replicates under a general reversible model with gamma distribution (GTR+G). The FigTree (v.1.4.2) program was used for visualization and printing of phylogenetic trees.

### Nucleotide sequence accession numbers

The nucleotide sequences obtained in this study are available from GenBank under accession numbers MK070525 to MK070532 and MK071345 to MK071456.

## Electronic supplementary material

Supplementary Figure Legends 1-21

Supplementary Table 1

Supplementary Table 2

Supplementary Table 3

Supplementary Table 4

Supplementary Table 5

Supplementary Table 6

Supplementary Figure 1

Supplementary Figure 2

Supplementary Figure 3

Supplementary Figure 4

Supplementary Figure 5

Supplementary Figure 6

Supplementary Figure 7

Supplementary Figure 8

Supplementary Figure 9

Supplementary Figure 10

Supplementary Figure 11

Supplementary Figure 12

Supplementary Figure 13

Supplementary Figure 14

Supplementary Figure 15

Supplementary Figure 16

Supplementary Figure 17

Supplementary Figure 18

Supplementary Figure 19

Supplementary Figure 20

Supplementary Figure 21
