# Supplementary material for: Evidence of a fixed internal gene constellation in influenza A viruses isolated from wild birds in Argentina (2006–2016)
Source: Emerg Microbes Infect. 2018 Nov 28;7:194. doi: 10.1038/s41426-018-0190-2 (PMC6258671; doi:10.1038/s41426-018-0190-2)
Supplement: Supplementary file 1 — Supplementary Figure Legends 1-21 [file 41426_2018_190_MOESM1_ESM.pdf]

Supplementary Figure Legends

SFig. 1. Maximum clade credibility tree inferred under a host-specific local clock model for IAVs internal gene segment PB2 (435 sequences). Color-coded annotations are shown by colored rectangles: South American (light red), Eurasian (light green) and North American (light blue). Viruses isolated from avian species are shown in colors depending on region of origin: South America (red), Eurasian (green), North America (blue), and Antarctica (violet). IAVs isolated from non-avian hosts are also shown in color depending on the host: Human (black), swine (pink), equine (brown) and bat (grey). Please note that IAV strains from avian hosts obtained from various South American countries are shown in red irrespective of their lineage. Posterior probabilities are shown. The asterisks (*) indicate the 15 IAVs isolates from Argentina analyzed in this study.

SFig. 2. Maximum clade credibility tree inferred under a host-specific local clock model for IAVs internal gene segment PB1 (413 sequences). Color-coded annotations are the same as described in SFig. 1. Posterior probabilities are shown. *IAVs isolates from Argentina obtained from this study.

SFig. 3. Maximum clade credibility tree inferred under a host-specific local clock model for IAVs internal gene segment PA (462 sequences). Color-coded annotations are the same as described in SFig. 1. Posterior probabilities are shown. *IAVs isolates from Argentina obtained from this study.

SFig. 4. Maximum clade credibility tree inferred under a host-specific local clock model for IAVs internal gene segment NP (508 sequences). Color-coded annotations are the same as described in SFig. 1. Posterior probabilities are shown. *IAVs isolates from Argentina obtained from this study.

SFig. 5. Maximum clade credibility tree inferred under a host-specific local clock model for IAVs internal gene segment M (519 sequences). Color-coded annotations are the same as described in SFig. 1. Posterior probabilities are shown. *IAVs isolates from Argentina obtained from this study.

SFig. 6. Maximum clade credibility tree inferred under a host-specific local clock model for IAVs internal gene segment NS (442 sequences). Color-coded annotations are the same as described in SFig. 1. Posterior probabilities are shown. *IAVs isolates from Argentina obtained from this study.

SFig. 7. Maximum likelihood phylogenetic tree of H1 gene segment (172 sequences). Color-coded annotations are the same as described in SFig. 1. Bootstrap values from 1000 replicates are shown. *IAVs isolates from Argentina obtained from this study.

SFig. 8. Maximum likelihood phylogenetic tree of H5 gene segment (183 sequences). Color-coded annotations are the same as described in SFig. 1. Bootstrap values from 1000 replicates are shown. *IAVs isolates from Argentina obtained from this study.

SFig. 9. Maximum likelihood phylogenetic tree of H7 gene segment (173 sequences). Color-coded annotations are the same as described in SFig. 1. Bootstrap values from 1000 replicates are shown. *IAVs isolates from Argentina obtained from this study.

SFig. 10. Maximum clade credibility tree inferred under an uncorrelated lognormal clock model for H10 gene segment (258 sequences). Color-coded annotations are the same as described in SFig. 1. Posterior probabilities are shown. *IAVs isolates from Argentina obtained from this study.

SFig. 11. Maximum clade credibility tree inferred under a host-specific local clock model for H4 gene segment (264 sequences). Color-coded annotations are the same as described in SFig. 1. Posterior probabilities are shown. *IAVs isolates from Argentina obtained from this study.

SFig. 12. Maximum clade credibility tree inferred under a host-specific local clock model for H6 gene segment (469 sequences). Color-coded annotations are the same as described in SFig. 1. The two clades containing viruses from Argentina are denoted as 1 and 2. Posterior probabilities are shown. *IAVs isolates from Argentina obtained from this study.

SFig. 13. Maximum likelihood phylogenetic tree of N1 gene segment (339 sequences). Color-coded annotations are the same as described in SFig. 1. Bootstrap values from 1000 replicates are shown. *IAVs isolates from Argentina obtained from this study.

SFig. 14. Maximum likelihood phylogenetic tree of N2 gene segment (637 sequences). Color-coded annotations are the same as described in SFig. 1. Bootstrap values from 1000 replicates are shown. *IAVs isolates from Argentina obtained from this study.

SFig. 15. Maximum likelihood phylogenetic tree of N3 gene segment (379 sequences). Color-coded annotations are the same as described in SFig. 1. Bootstrap values from 1000 replicates are shown. *IAVs isolates from Argentina obtained from this study.

SFig. 16. Maximum likelihood phylogenetic tree of N6 gene segment (264 sequences). Color-coded annotations are the same as described in SFig. 1. Bootstrap values from 1000 replicates are shown. *IAVs isolates from Argentina obtained from this study.

SFig. 17. Maximum likelihood phylogenetic tree of N9 gene segment (297 sequences). Color-coded annotations are the same as described in SFig. 1. Bootstrap values from 1000 replicates are shown. *IAVs isolates from Argentina obtained from this study.

SFig. 18. Maximum clade credibility tree inferred under a host-specific local clock model for N7 gene segment (213 sequences). Color-coded annotations are the same as described in SFig. 1. Posterior probabilities are shown. *IAVs isolates from Argentina obtained from this study.

SFig. 19. Maximum clade credibility trees inferred under a host-specific local clock model for N8 gene segment (463 sequences). Color-coded annotations are the same as described in SFig. 1. Posterior probabilities are shown. *IAVs isolates from Argentina obtained from this study.

SFig. 20. Alignment of PB1-F2 predicted amino acid sequences from Argentinean LPAIV isolated from 2006 to 2016.

SFig. 21. Alignment of PA-X predicted amino acid sequences from Argentinean LPAIV isolated from 2006 to 2016.
