# Supplementary material for: Evidence of a fixed internal gene constellation in influenza A viruses isolated from wild birds in Argentina (2006–2016)
Source: Emerg Microbes Infect. 2018 Nov 28;7:194. doi: 10.1038/s41426-018-0190-2 (PMC6258671; doi:10.1038/s41426-018-0190-2)
Supplement: Supplementary file 2 — Supplementary Table 1 [file 41426_2018_190_MOESM2_ESM.doc]

STable 1. Number of cloacal swabs and avian species sampled for IAV detection in Argentina from 2006 to 2016.

| **Order** | **Family** | **Scientific name** | **English name** | **2006** | **2007** | **2008** | **2009** | **2010** | **2011** | **2012** | **2013** | **2014** | **2015** | **2016** | **Total** |
| --- | --- | --- | --- | --- | --- | --- | --- | --- | --- | --- | --- | --- | --- | --- | --- |
| Accipitriformes | Accipitridae | *Buteogallus coronatus* | Chaco Eagle | 0 | 0 | 0 | 0 | 0 | 0 | 16 | 0 | 0 | 0 | 0 | 16 |
| Anseriformes | Anatidae | *Netta peposaca* | Rosy-billed Pochard | 157 | 615 | 122 | 185 | 86 | 100 | 0 | 0 | 0 | 0 | 132 | 1397 |
| Anseriformes | Anatidae | *Dendrocygna viduata* | Black-bellied Whistling-Duck | 30 | 1 | 142 | 26 | 0 | 98 | 73 | 0 | 0 | 0 | 70 | 440 |
| Anseriformes | Anatidae | *Anas versicolor* | Silver Teal | 24 | 9 | 42 | 156 | 11 | 139 | 0 | 0 | 0 | 0 | 0 | 381 |
| Anseriformes | Anatidae | *Dendrocygna bicolor* | White-faced Whistling-Duck | 78 | 25 | 43 | 13 | 22 | 104 | 0 | 0 | 0 | 0 | 25 | 310 |
| Anseriformes | Anatidae | *Amazonetta brasiliensis* | Brazilian Teal | 52 | 53 | 18 | 15 | 5 | 18 | 32 | 0 | 0 | 0 | 54 | 247 |
| Anseriformes | Anatidae | *Anas platalea* | Red Shoveler | 4 | 1 | 8 | 132 | 2 | 8 | 0 | 0 | 0 | 0 | 1 | 156 |
| Anseriformes | Anatidae | *Callonetta leucophrys* | Ringed Teal | 23 | 38 | 11 | 29 | 8 | 37 | 0 | 0 | 0 | 0 | 6 | 152 |
| Anseriformes | Anatidae | *Anas flavirostris* | Yellow-billed Teal | 11 | 5 | 0 | 1 | 2 | 3 | 0 | 0 | 0 | 0 | 35 | 57 |
| Anseriformes | Anatidae | *Anas georgica* | Yellow-billed Pintail | 3 | 1 | 0 | 0 | 0 | 6 | 0 | 0 | 0 | 0 | 20 | 30 |
| Anseriformes | Anatidae | *Anas bahamensis* | White-cheeked Pintail | 0 | 5 | 0 | 2 | 19 | 1 | 0 | 0 | 0 | 0 | 1 | 28 |
| Anseriformes | Anatidae | *Dendrocygna autumnalis* | Fulvous Whistling-Duck | 0 | 0 | 0 | 0 | 0 | 5 | 20 | 0 | 0 | 0 | 0 | 25 |
| Anseriformes | Anatidae | *Sarkidiornis melanotos* | Comb Duck | 0 | 1 | 10 | 0 | 0 | 1 | 0 | 0 | 0 | 0 | 0 | 12 |
| Anseriformes | Anatidae | *Anas cyanoptera* | Cinnamon Teal | 0 | 0 | 1 | 9 | 0 | 1 | 0 | 0 | 0 | 0 | 0 | 11 |
| Anseriformes | Anatidae | *Heteronetta atricapilla* | Black-headed Duck | 0 | 1 | 1 | 5 | 0 | 0 | 0 | 0 | 0 | 0 | 0 | 7 |
| Anseriformes | Anatidae | *Anas sibilatrix* | Chiloe Wigeon | 0 | 0 | 0 | 0 | 1 | 0 | 0 | 0 | 0 | 0 | 1 | 2 |
| Anseriformes | Anatidae | *Coscoroba coscoroba* | Coscoroba Swan | 1 | 0 | 0 | 0 | 0 | 0 | 0 | 0 | 0 | 0 | 0 | 1 |
| Anseriformes | Anatidae | *Nomonyx dominicus* | Masked Duck | 1 | 0 | 0 | 0 | 0 | 0 | 0 | 0 | 0 | 0 | 0 | 1 |
| Anseriformes | Anhimidae | *Chauna torquata* | Southern Screamer | 0 | 0 | 1 | 0 | 0 | 0 | 0 | 0 | 0 | 0 | 0 | 1 |
| Cathartiformes | Cathartidae | *Vultur gryphus* | Andean Condor | 0 | 0 | 0 | 0 | 0 | 0 | 49 | 0 | 0 | 0 | 0 | 49 |
| Charadriiformes | Laridae | *Sterna hirundo* | Common Tern | 9 | 10 | 42 | 0 | 0 | 0 | 0 | 0 | 76 | 341 | 704 | 1182 |
| Charadriiformes | Laridae | *Larus dominicanus* | Kelp Gull | 114 | 60 | 38 | 6 | 0 | 20 | 0 | 0 | 0 | 0 | 0 | 238 |
| Charadriiformes | Scolopacidae | *Calidris canutus* | Red Knot | 0 | 0 | 0 | 29 | 0 | 0 | 0 | 0 | 76 | 0 | 0 | 105 |
| Charadriiformes | Charadriidae | *Charadrius falklandicus* | Two-banded Plover | 0 | 0 | 0 | 73 | 0 | 0 | 0 | 0 | 29 | 0 | 0 | 102 |
| Charadriiformes | Laridae | *Thalasseus sandvicensis* | Sandwich Tern | 9 | 40 | 47 | 0 | 0 | 0 | 0 | 0 | 0 | 0 | 1 | 97 |
| Charadriiformes | Scolopacidae | *Calidris fuscicollis* | White-rumped Sandpiper | 0 | 3 | 0 | 7 | 0 | 0 | 0 | 0 | 84 | 0 | 0 | 94 |
| Charadriiformes | Laridae | *Sterna hirundinacea* | South American Tern | 17 | 40 | 15 | 0 | 0 | 0 | 0 | 0 | 0 | 0 | 14 | 86 |
| Charadriiformes | Laridae | *Thalasseus maximus* | Royal Tern | 7 | 18 | 19 | 0 | 0 | 0 | 0 | 0 | 0 | 0 | 0 | 44 |
| Charadriiformes | Rynchopidae | *Rynchops niger* | Black Skimmer | 0 | 6 | 0 | 0 | 0 | 0 | 0 | 0 | 0 | 0 | 0 | 6 |
| Charadriiformes | Scolopacidae | *Gallinago paraguaiae* | South American Snipe | 0 | 0 | 1 | 5 | 0 | 0 | 0 | 0 | 0 | 0 | 0 | 6 |
| Charadriiformes | Laridae | *Chroicocephalus cirrocephalus* | Gray-hooded Gull | 0 | 0 | 0 | 0 | 0 | 0 | 0 | 0 | 0 | 0 | 3 | 3 |
| Charadriiformes | Rostratulidae | *Nycticryphes semicollaris* | South American Painted-snipe | 0 | 0 | 3 | 0 | 0 | 0 | 0 | 0 | 0 | 0 | 0 | 3 |
| Charadriiformes | Charadriidae | *Vanellus chilensis* | Southern Lapwing | 0 | 0 | 2 | 0 | 0 | 0 | 0 | 0 | 0 | 0 | 0 | 2 |
| Charadriiformes | Laridae | *Chroicocephalus maculipennis* | Brown-hooded Gull | 0 | 0 | 0 | 0 | 0 | 0 | 0 | 0 | 0 | 0 | 2 | 2 |
| Charadriiformes | Laridae | *Sterna trudeaui* | Snowy-crowned Tern | 0 | 0 | 1 | 0 | 0 | 0 | 0 | 0 | 0 | 0 | 0 | 1 |
| Charadriiformes | Scolopacidae | *Phalaropus tricolor* | Wilson's Phalarope | 0 | 0 | 1 | 0 | 0 | 0 | 0 | 0 | 0 | 0 | 0 | 1 |
| Charadriiformes | Scolopacidae | *Tringa flavipes* | Lesser Yellowlegs | 0 | 0 | 1 | 0 | 0 | 0 | 0 | 0 | 0 | 0 | 0 | 1 |
| Columbiformes | Columbidae | *Columbina picui* | Picui Ground Dove | 11 | 0 | 0 | 0 | 0 | 0 | 0 | 0 | 0 | 0 | 0 | 11 |
| Columbiformes | Columbidae | *Patagioenas maculosa* | Spot-winged Pigeon | 3 | 0 | 0 | 0 | 0 | 0 | 0 | 0 | 0 | 0 | 0 | 3 |
| Columbiformes | Columbidae | *Patagioenas picazuro* | Picazuro Pigeon | 0 | 0 | 0 | 0 | 0 | 0 | 0 | 0 | 0 | 0 | 2 | 2 |
| Gruiformes | Rallidae | *Fulica leucoptera* | White-winged Coot | 13 | 3 | 0 | 0 | 0 | 0 | 0 | 0 | 0 | 0 | 0 | 16 |
| Gruiformes | Rallidae | *Fulica rufifrons* | Red-fronted Coot | 1 | 0 | 0 | 0 | 0 | 0 | 0 | 0 | 0 | 0 | 0 | 1 |
| Gruiformes | Rallidae | *Pardirallus maculatus* | Spotted Rail | 0 | 0 | 1 | 0 | 0 | 0 | 0 | 0 | 0 | 0 | 0 | 1 |
| Passeriformes | Turdidae | *Turdus amaurochalinus* | Creamy-bellied Thrush | 10 | 0 | 0 | 0 | 0 | 0 | 0 | 0 | 0 | 0 | 0 | 10 |
| Passeriformes | Furnariidae | *Furnarius rufus* | Rufous Hornero | 3 | 0 | 0 | 0 | 0 | 0 | 0 | 0 | 0 | 0 | 0 | 3 |
| Passeriformes | Furnariidae | *Synallaxis frontalis* | Sooty-fronted Spinetail | 2 | 0 | 0 | 0 | 0 | 0 | 0 | 0 | 0 | 0 | 0 | 2 |
| Passeriformes | Icteridae | *Molothrus bonariensis* | Shiny Cowbird | 2 | 0 | 0 | 0 | 0 | 0 | 0 | 0 | 0 | 0 | 0 | 2 |
| Passeriformes | Polioptilidae | *Polioptila dumicola* | Masked Gnatcatcher | 2 | 0 | 0 | 0 | 0 | 0 | 0 | 0 | 0 | 0 | 0 | 2 |
| Passeriformes | Turdidae | *Turdus rufiventris* | Rufous-bellied Thrush | 2 | 0 | 0 | 0 | 0 | 0 | 0 | 0 | 0 | 0 | 0 | 2 |
| Passeriformes | Emberizidae | *Zonotrichia capensis* | Rufous-collared Sparrow | 1 | 0 | 0 | 0 | 0 | 0 | 0 | 0 | 0 | 0 | 0 | 1 |
| Passeriformes | Thamnophilidae | *Taraba major* | Great Antshrike | 1 | 0 | 0 | 0 | 0 | 0 | 0 | 0 | 0 | 0 | 0 | 1 |
| Passeriformes | Thraupidae | *Saltator coerulescens* | Grayish Saltator | 1 | 0 | 0 | 0 | 0 | 0 | 0 | 0 | 0 | 0 | 0 | 1 |
| Pelecaniformes | Threskiornithidae | *Plegadis chihi* | White-faced Ibis | 5 | 0 | 0 | 0 | 0 | 0 | 0 | 0 | 0 | 0 | 0 | 5 |
| Pelecaniformes | Ardeidae | *Ardea alba* | Great Egret | 1 | 0 | 0 | 0 | 0 | 0 | 0 | 0 | 0 | 0 | 0 | 1 |
| Pelecaniformes | Ardeidae | *Ardea cocoi* | Cocoi Heron | 1 | 0 | 0 | 0 | 0 | 0 | 0 | 0 | 0 | 0 | 0 | 1 |
| Pelecaniformes | Threskiornithidae | *Phimosus infuscatus* | Bare-faced Ibis | 1 | 0 | 0 | 0 | 0 | 0 | 0 | 0 | 0 | 0 | 0 | 1 |
| Procellariiformes | Procellariidae | *Macronectes giganteus* | Southern Giant-Petrel | 0 | 18 | 0 | 0 | 0 | 0 | 0 | 0 | 0 | 0 | 0 | 18 |
| Psittaciformes | Psittacidae | *Amazona aestiva* | Turquoise-fronted Parrot | 60 | 0 | 0 | 0 | 0 | 0 | 0 | 0 | 0 | 0 | 0 | 60 |
| Sphenisciformes | Spheniscidae | *Spheniscus magellanicus* | Magellanic Penguin | 91 | 310 | 68 | 0 | 0 | 0 | 210 | 65 | 0 | 0 | 0 | 744 |
| Suliformes | Phalacrocoracidae | *Phalacrocorax atriceps* | Imperial Cormorant | 17 | 157 | 90 | 0 | 0 | 65 | 0 | 0 | 0 | 0 | 0 | 329 |
| Suliformes | Phalacrocoracidae | *Phalacrocorax magellanicus* | Magellanic Cormorant | 1 | 1 | 54 | 0 | 0 | 0 | 0 | 0 | 0 | 0 | 0 | 56 |
| Suliformes | Phalacrocoracidae | *Phalacrocorax brasilianus* | Neotropic Cormorant | 1 | 0 | 0 | 0 | 0 | 0 | 0 | 0 | 0 | 0 | 25 | 26 |
| **Total** |  |  |  | 770 | 1421 | 782 | 693 | 156 | 606 | 400 | 65 | 265 | 341 | 1096 | 6595 |
