# Supplementary material for: Evidence of a fixed internal gene constellation in influenza A viruses isolated from wild birds in Argentina (2006–2016)
Source: Emerg Microbes Infect. 2018 Nov 28;7:194. doi: 10.1038/s41426-018-0190-2 (PMC6258671; doi:10.1038/s41426-018-0190-2)
Supplement: Supplementary file 3 — Supplementary Table 2 [file 41426_2018_190_MOESM3_ESM.doc]

STable 2. Number of RT-qPCR positive samples and isolates obtained for different types of avian species sampled in Argentina from 2006 to 2016.

| Order | Family | Scientific name | English name | Total samples | Number of positive RRT-PCR | Isolate subtypes (number of isolates) |
| --- | --- | --- | --- | --- | --- | --- |
| Anseriformes | Anatidae | *Netta peposaca* | Rosy-billed Pochard | 1397 | 16 | H6N2 (4), H6N8 (2), H9N2 (1) |
| Anseriformes | Anatidae | *Anas versicolor* | Silver Teal | 381 | 23 | H4N2 (1), H4N6 (1), H4N8 (1), H5N3 (1), H6N2 (1), H7N7 (1), H10N7 (2) |
| Anseriformes | Anatidae | *Amazonetta brasiliensis* | Brazilian Teal | 247 | 5 |  |
| Anseriformes | Anatidae | *Anas platalea* | Red Shoveler | 156 | 1 |  |
| Anseriformes | Anatidae | *Callonetta leucophrys* | Ringed Teal | 152 | 1 |  |
| Anseriformes | Anatidae | *Anas flavirostris* | Yellow-billed Teal | 57 | 2 | H4N6 (2) |
| Anseriformes | Anatidae | *Anas georgica* | Yellow-billed Pintail | 30 | 1 | H6N2 (1) |
| Anseriformes | Anatidae | *Anas bahamensis* | White-cheeked Pintail | 28 | 1 |  |
| Anseriformes | Anatidae | *Sarkidiornis melanotos* | Comb Duck | 12 | 1 | H6N2 (1) |
| Anseriformes | Anatidae | *Anas cyanoptera* | Cinnamon Teal | 11 | 2 | H1N1 (1), H7N9 (1) |
| Charadriiformes | Laridae | *Larus dominicanus* | Kelp Gull | 238 | 1 | H13N9 (1) |
| Psittaciformes | Psittacidae | *Amazona aestiva* | Turquoise-fronted Parrot | 60 | 1 |  |
| Total |  |  |  |  | 55 | 22 |
|  | | | |  |  |  |
