# Supplementary material for: Evidence of a fixed internal gene constellation in influenza A viruses isolated from wild birds in Argentina (2006–2016)
Source: Emerg Microbes Infect. 2018 Nov 28;7:194. doi: 10.1038/s41426-018-0190-2 (PMC6258671; doi:10.1038/s41426-018-0190-2)
Supplement: Supplementary file 4 — Supplementary Table 3 [file 41426_2018_190_MOESM4_ESM.doc]

STable 3. Avian-origin influenza virus isolates collected from 2006 to 2016.

| Strain Name | Subtype | Short name | Host species name | English name |  |
| --- | --- | --- | --- | --- | --- |
| A/cinnamon teal/Argentina/CIP051-432/2011 (H1N1)* | H1N1 | 432/H1N1 | *Anas cyanoptera* | cinnamon teal |  |
| A/silver teal/Argentina/CIP051-32/2011 (H4N2)*^#^ | H4N2 | 32/H4N2 | *Anas versicolor* | silver teal |  |
| A/silver teal/Argentina/CIP051-48/2011 (H4N6)*^#^ | H4N6 | 48/H4N6 | *Anas versicolor* | silver teal |  |
| A/yellow-billed teal/Argentina/CIP051-91/2011 (H4N6)* | H4N6 | 91/H4N6 | *Anas flavirostris* | yellow-billed teal |  |
| A/yellow-billed teal/Argentina/CIP112-1227/2016 (H4N6)* | H4N6 | 1227/H4N6 | *Anas flavirostris* | yellow-billed teal |  |
| A/silver teal/Argentina/CIP051-25/2011 (H4N8)*^#^ | H4N8 | 25/H4N8 | *Anas versicolor* | silver teal |  |
| A/silver teal/Argentina/CIP051-1737/2009 (H5N3)* | H5N3 | 1737/H5N3 | *Anas versicolor* | silver teal |  |
| A/rosy-billed pochard/Argentina/CIP051-272/2007 (H6N2)*^#^ | H6N2 | 272/H6N2 | *Netta peposaca* | rosy-billed pochard |  |
| A/rosy-billed pochard/Argentina/CIP051-557/2007 (H6N2)^a#^ | H6N2 | 557/H6N2 | *Netta peposaca* | rosy-billed pochard |  |
| A/rosy-billed pochard/Argentina/CIP051-925/2008 (H6N2)^a#^ | H6N2 | 925/H6N2 | *Netta peposaca* | rosy-billed pochard |  |
| A/rosy-billed pochard/Argentina/CIP051-1977/2010 (H6N2)^a^ | H6N2 | 1977/H6N2 | *Netta peposaca* | rosy-billed pochard |  |
| A/comb duck/Argentina/CIP051-49/2011 (H6N2)* | H6N2 | 49/H6N2 | *Sarkidiornis melanotos* | comb duck |  |
| A/silver teal/Argentina/CIP051-52/2011 (H6N2)* | H6N2 | 52/H6N2 | *Anas versicolor* | silver teal |  |
| A/yellow-billed pintail/Argentina/CIP112-1174A/2016 (H6N2)* | H6N2 | 1174A/H6N2 | *Anas georgica* | yellow-billed pintail |  |
| A/rosy-billed pochard/Argentina/CIP051-269/2007 (H6N8)^a#^ | H6N8 | 269/H6N8 | *Netta peposaca* | rosy-billed pochard |  |
| A/rosy-billed pochard/Argentina/CIP051-575/2007 (H6N8)^a#^ | H6N8 | 575/H6N8 | *Netta peposaca* | rosy-billed pochard |  |
| A/silver teal/Argentina/CIP051-188/2011 (H7N7)* | H7N7 | 188/H7N7 | *Anas versicolor* | silver teal |  |
| A/cinnamon teal/Argentina/CIP051-1588/2009 (H7N9)*^#^ | H7N9 | 1588/H7N9 | *Anas cyanoptera* | cinnamon teal |  |
| A/rosy-billed pochard/Argentina/CIP051-559/2007 (H9N2)^b#^ | H9N2 | 559/H9N2 | *Netta peposaca* | rosy-billed pochard |  |
| A/silver teal/Argentina/CIP051-171/2011 (H10N7)* | H10N7 | 171/H10N7 | *Anas versicolor* | silver teal |  |
| A/silver teal/Argentina/CIP051-175/2011 (H10N7)* | H10N7 | 175/H10N7 | *Anas versicolor* | silver teal |  |
| A/kelp gull/Argentina/CIP051-LDC4/2006 (H13N9)^c#^ | H13N9 | LDC4/H13N9 | *Larus dominicanus* | kelp gull |  |
|  |  |  |  |  |  |
| * New fifteen IAVs isolates from Argentina from this study | | | | | |
| ^a^ Rimondi A. et al., J Virol. 2011 Dec;85(24):13354-62. doi: 10.1128/JVI.05946-11 | | | | | |
| ^b^ Xu K. et al., Virus Res. 2012 Sep;168(1-2):41-7. doi: 10.1016/j.virusres.2012.06.010 | | | | | |
| ^c^ Pereda A. et al., Virology. 2008 Sep 1;378(2):363-70. doi: 10.1016/j.virol.2008.06.010 | | |  |  |  |
| ^#^ IAVs re-sequenced by Next Generation Sequencing |  |  |  |  |  |
