# Supplementary material for: Evidence of a fixed internal gene constellation in influenza A viruses isolated from wild birds in Argentina (2006–2016)
Source: Emerg Microbes Infect. 2018 Nov 28;7:194. doi: 10.1038/s41426-018-0190-2 (PMC6258671; doi:10.1038/s41426-018-0190-2)
Supplement: Supplementary file 5 — Supplementary Table 4 [file 41426_2018_190_MOESM5_ESM.doc]

STable 4. Estimated mean times of divergence of the main South American avian IAV clades. 95% credibility intervals are shown.

| Gene | TMRCA | 95% HPD | | Between |
| --- | --- | --- | --- | --- |
|  |  | Low | High |  |
|  |  |  |  |  |
| PB2 | 1886.9 | 1880.0 | 1893.6 | Eurasian avian and South American avian |
|  |  |  |  |  |
| PB1 | 1988.2 | 1986.8 | 1989.4 | North American avian and South American avian |
|  |  |  |  |  |
| PA | 1943.3 | 1938.6 | 1947.2 | Equine and Antarctica and South American avian |
|  |  |  |  |  |
| NP | 1951.1 | 1947.4 | 1955.1 | Equine and South American avian |
|  |  |  |  |  |
| M | 1971.4 | 1968.2 | 1974.3 | North American avian and South American avian |
|  |  |  |  |  |
| NS-A | 1948.0 | 1941.3 | 1953.9 | Equine and South American avian |
| NS-B | 1945.6 | 1938.5 | 1953.2 | North American avian and South American avian |
|  |  |  |  |  |
| H4 | 2008.1 | 2007.2 | 2008.5 | Introduction of North American avian H4 to Argentina |
|  |  |  |  |  |
| H6 | 1981.4 | 1978.7 | 1984.4 | First introduction of North American avian H6 to Argentina (lineage A) |
|  | 2003.5 | 2002.5 | 2004.0 | Second introduction of North American avian H6 to Argentina (lineage A) |
|  |  |  |  |  |
| H10 | 1941.1 | 1933.1 | 1949.4 | North American avian and South American avian |
|  |  |  |  |  |
| N7 | 1937.4 | 1932.2 | 1944.5 | North American avian and South American avian |
|  |  |  |  |  |
| N8 | 1978.3 | 1976.3 | 1980.3 | Introduction of North American avian N8 to Argentina |
|  |  |  |  |  |
