# Supplementary material for: Evidence of a fixed internal gene constellation in influenza A viruses isolated from wild birds in Argentina (2006–2016)
Source: Emerg Microbes Infect. 2018 Nov 28;7:194. doi: 10.1038/s41426-018-0190-2 (PMC6258671; doi:10.1038/s41426-018-0190-2)
Supplement: Supplementary file 6 — Supplementary Table 5 [file 41426_2018_190_MOESM6_ESM.doc]

STable 5. Internal gene segment constellations by host order of all South American LPAIV sequences (Argentinean and South American viruses included in MCC trees and sequences available at the Influenza Research Database). Lineage notation: South American (SAm), North American (NAm), Eurasian (EAs). Shorebird (Sh.) and Antarctica (An.) specific lineages are also indicated. N/D: No sequence data available. * New fifteen IAVs isolates from Argentina from this study.

| Host order | Name | Country | Subtype | PB2 | PB1 | PA | NP | M | NS |
| --- | --- | --- | --- | --- | --- | --- | --- | --- | --- |
| Anseriformes | A/rosy-billed_pochard/Argentina/CIP051-559/2007 | Argentina | H9N2 | SAm | SAm | SAm | SAm | SAm | SAm (a) |
| Anseriformes | A/rosy-billed_pochard/Argentina/CIP051-269/2007 | Argentina | H6N8 | SAm | SAm | SAm | SAm | SAm | SAm (b) |
| Anseriformes | A/rosy-billed_pochard/Argentina/CIP051-272/2007 | Argentina | H6N2* | SAm | SAm | SAm | SAm | SAm | SAm (b) |
| Anseriformes | A/rosy-billed_pochard/Argentina/CIP051-557/2007 | Argentina | H6N2 | SAm | SAm | SAm | SAm | SAm | SAm (b) |
| Anseriformes | A/rosy-billed_pochard/Argentina/CIP051-575/2007 | Argentina | H6N8 | SAm | SAm | SAm | SAm | SAm | SAm (b) |
| Anseriformes | A/rosy-billed_pochard/Argentina/CIP051-925/2008 | Argentina | H6N2 | SAm | SAm | SAm | SAm | SAm | SAm (a) |
| Anseriformes | A/cinnamon_teal/Argentina/CIP051-1588/2009 | Argentina | H7N9* | SAm | SAm | SAm | SAm | SAm | SAm (a) |
| Anseriformes | A/silver_teal/Argentina/CIP051-1737/2009 | Argentina | H5N3* | SAm | SAm | SAm | SAm | SAm | SAm (a) |
| Anseriformes | A/rosy-billed_pochard/Argentina/CIP051-1977/2010 | Argentina | H6N2 | SAm | SAm | SAm | SAm | SAm | SAm (a) |
| Anseriformes | A/cinnamon_teal/Argentina/CIP051-432/2011 | Argentina | H1N1* | SAm | SAm | SAm | SAm | SAm | SAm (a) |
| Anseriformes | A/comb_duck/Argentina/CIP051-49/2011 | Argentina | H6N2* | SAm | SAm | SAm | SAm | SAm | SAm (a) |
| Anseriformes | A/silver_teal/Argentina/CIP051-171/2011 | Argentina | H10N7* | SAm | SAm | SAm | SAm | SAm | SAm (a) |
| Anseriformes | A/silver_teal/Argentina/CIP051-175/2011 | Argentina | H10N7* | SAm | SAm | SAm | SAm | SAm | SAm (a) |
| Anseriformes | A/silver_teal/Argentina/CIP051-25/2011 | Argentina | H4N8* | SAm | SAm | SAm | SAm | SAm | SAm (a) |
| Anseriformes | A/silver_teal/Argentina/CIP051-32/2011 | Argentina | H4N2* | SAm | SAm | SAm | SAm | SAm | SAm (a) |
| Anseriformes | A/silver_teal/Argentina/CIP051-52/2011 | Argentina | H6N2* | SAm | SAm | SAm | SAm | SAm | SAm (a) |
| Anseriformes | A/silver-teal/Argentina/CIP051-48/2011 | Argentina | H4N6* | SAm | SAm | SAm | SAm | SAm | SAm (a) |
| Anseriformes | A/yellow-billed_teal/Argentina/CIP051-91/2011 | Argentina | H4N6* | SAm | SAm | SAm | SAm | SAm | SAm (a) |
| Anseriformes | A/silver_teal/Argentina/CIP051-188/2011 | Argentina | H7N7* | SAm | SAm | SAm | SAm | SAm | SAm (b) |
| Anseriformes | A/yellow-billed_pintail/Argentina/CIP112-1174A/2016_ | Argentina | H6N2* | SAm | SAm | SAm | SAm | SAm | SAm (a) |
| Anseriformes | A/yellow-billed_teal/Argentina/CIP112-1227/2016 | Argentina | H4N6* | SAm | SAm | SAm | SAm | SAm | SAm (a) |
| Anseriformes | A/cinnamon_teal/Bolivia/4537/2001 | Bolivia | H7N3 | SAm | SAm | SAm | SAm | NAm | SAm (a) |
| Anseriformes | A/white_rumped_sandpiper/Lagoa_do_Peixe/RS1167/2012 | Brazil | H6N1 | SAm | SAm | NAm | SAm | N/D | NAm (a) |
| Anseriformes | A/yellow_billed_pintail/Chile/1/2012 | Chile | H1N1 | SAm | NAm | SAm | SAm | SAm | SAm (b) |
| Anseriformes | A/yellow_billed_teal/Chile/8/2013 | Chile | H7N6 | N/D | N/D | SAm | SAm | SAm | SAm (b) |
| Anseriformes | A/Chilean_teal/Chile/9/2013 | Chile | H7N6 | SAm | SAm | N/D | SAm | SAm | NAm (b) |
| Anseriformes | A/Chilean_teal/Chile/8/2013 | Chile | H7N6 | SAm | SAm | N/D | SAm | SAm | SAm (b) |
| Anseriformes | A/yellow_billed_pintail/Chile/6/2013 | Chile | H4N6 | SAm | SAm | SAm | SAm | SAm | SAm (a) |
| Anseriformes | A/yellow_billed_pintail/Chile/7/2013 | Chile | H4N6 | SAm | SAm | SAm | SAm | SAm | SAm (a) |
| Anseriformes | A/Chilean_teal/Chile/12/2014 | Chile | H7N3 | SAm | SAm | SAm | N/D | N/D | SAm (b) |
| Anseriformes | A/yellow_billed_pintail/Chile/10/2014 | Chile | H7N3 | SAm | SAm | SAm | SAm | SAm | SAm (a) |
| Anseriformes | A/yellow_billed_pintail/Chile/11/2014 | Chile | H7N3 | SAm | SAm | SAm | SAm | SAm | SAm (a) |
| Anseriformes | A/yellow_billed_teal/Chile/12/2014 | Chile | H7N4 | SAm | SAm | SAm | SAm | SAm | SAm (b) |
| Anseriformes | A/yellow_billed_pintail/Chile/C1267/2015 | Chile | H5N3 | SAm | NAm | SAm | SAm | SAm | SAm (a) |
| Anseriformes | A/Chilean_teal/Chile/C918/2015 | Chile | H4N2 | SAm | SAm | SAm | SAm | SAm | SAm (a) |
| Anseriformes | A/mallard/Chile/C948/2015 | Chile | H4N2 | SAm | SAm | SAm | SAm | SAm | SAm (a) |
| Anseriformes | A/black_bellied_whistling_duck/Colombia/1/2011 | Colombia | H5N2 | NAm | NAm | NAm (a) | NAm | NAm | NAm (a) |
| Anseriformes | A/white_faced_whistling_duck/Colombia/1/2011 | Colombia | H5N2 | NAm | NAm | NAm (a) | NAm | NAm | NAm (a) |
| Anseriformes | A/duck/Peru/32/2006 | Peru | H3N8 | NAm | NAm | NAm (a) | NAm | NAm (a) | NAm (b) |
| Anseriformes | A/duck/Peru_MM23/2007 | Peru | H4N5 | NAm | NAm | NAm (a) | NAm | NAm | NAm (b) |
| Anseriformes | A/duck/Peru/MM17/2007 | Peru | H4N5 | NAm | NAm | NAm (a) | NAm | NAm | NAm (b) |
| Anseriformes | A/duck/Peru_PV72/2008 | Peru | H7N3 | NAm | NAm | NAm | NAm | NAm | NAm (a) |
| Anseriformes | A/duck/Peru/PV80/2008 | Peru | H2N9 | NAm | NAm | NAm (a) | NAm | NAm | NAm (b) |
| Anseriformes | A/duck/Peru/P114/2009 | Peru | H3N2 | NAm | NAm | NAm | NAm | NAm | NAm (a) |
| Anseriformes | A/duck/Peru_PuV196/2009 | Peru | H10N2 | NAm | NAm | N/D | NAm | NAm | NAm (a) |
| Anseriformes | A/duck/Peru/CH36/2010 | Peru | H11N9 | NAm | NAm | NAm (a) | NAm | NAm | NAm (a) |
| Avian | A/wild_bird/Chile/1805/2008 | Chile | H5N9 | NAm | NAm | NAm | NAm | NAm | NAm (a) |
| Charadriiformes | A/kelp_gull/Argentina/CIP051-LDC4/2006 | Argentina | H13N9 | SAm | SAm | SAm | SAm | SAm | SAm (a) |
| Charadriiformes | A/semi-palmated_sandpiper/Brazil/43/1990 | Brazil | H2N1 | NAm | NAm | NAm | SAm | SAm | SAm (a) |
| Charadriiformes | A/ruddy_turnstone/Ilha_de_Canelas/A08/2008 | Brazil | H11N9 | NAm | Sh. | NAm | NAm | NAm | NAm (a) |
| Charadriiformes | A/ruddy_turnstone/Ilha_de_Canelas/A17/2008 | Brazil | H11N9 | NAm | Sh. | NAm | NAm | NAm | NAm (a) |
| Charadriiformes | A/ruddy_turnstone/Ilha_de_Canelas/A51/2008 | Brazil | H11N9 | NAm | Sh. | NAm | NAm | NAm | NAm (a) |
| Charadriiformes | A/white_rumped_sandpiper/Lagoa_do_Peixe/RS1151/2012 | Brazil | H6N1 | SAm | SAm | NAm | SAm | SAm | NAm (a) |
| Charadriiformes | A/white_rumped_sandpiper/Lagoa_do_Peixe/RS1154/2012 | Brazil | H6N1 | SAm | SAm | NAm | SAm | SAm | NAm (a) |
| Charadriiformes | A/white_rumped_sandpiper/Lagoa_do_Peixe/RS1169/2012 | Brazil | H6N1 | SAm | SAm | NAm | SAm | SAm | NAm (a) |
| Charadriiformes | A/white_rumped_sandpiper/Lagoa_do_Peixe/RS1177/2012 | Brazil | H6N1 | SAm | SAm | NAm | SAm | SAm | NAm (a) |
| Charadriiformes | A/white_rumped_sandpiper/Lagoa_do_Peixe/RS1196/2012 | Brazil | H6N1 | SAm | SAm | NAm | SAm | SAm | NAm (a) |
| Charadriiformes | A/seagull/Chile/5775/2009 | Chile | H13N9 | EAs (Sh.) | NAm | Sh. | Sh. | EAs (Sh.) | Sh. (a) |
| Charadriiformes | A/black_necked_stilt/Chile/1/2013 | Chile | H11N9 | NAm | NAm | NAm | NAm | NAm | NAm (a) |
| Charadriiformes | A/black_necked_stilt/Chile/2/2013 | Chile | H11N9 | NAm | NAm | NAm | NAm | NAm | NAm (a) |
| Charadriiformes | A/gray_plover/Chile/C1313/2015 | Chile | H9N7 | NAm | NAm | NAm (a) | SAm | SAm | NAm (a) |
| Charadriiformes | A/American_oystercatcher/Chile/C1307/2015 | Chile | H9N2 | NAm | SAm | NAm (a) | SAm | SAm | NAm (a) |
| Charadriiformes | A/ruddy_turnstone/Peru/XVII/2006 | Peru | H10N9 | NAm | NAm | NAm (a) | NAm | SAm | NAm (a) |
| Charadriiformes | A/oystercatcher/Peru/34/2006 | Peru | H10N9 | NAm | NAm | N/D | NAm | NAm | NAm (a) |
| Charadriiformes | A/whimbrel/Peru/P41/2007 | Peru | H13N2 | NAm | Peru | EAs | Sh. | EAs (Sh.) | Sh. (a) |
| Charadriiformes | A/gull/Peru/P43/2007 | Peru | H13N2 | NAm | Peru | EAs | Sh. | EAs (Sh.) | Sh. (a) |
| Charadriiformes | A/ruddy_turnstone/Peru/MM149/2008 | Peru | H10N7 | NAm | NAm | NAm | NAm | NAm | NAm (a) |
| Charadriiformes | A/ruddy_turnstone/Peru/PuV51/2008 | Peru | H12N5 | NAm | NAm | NAm (a) | NAm | NAm | NAm (a) |
| Charadriiformes | A/ruddy_turnstone/Peru/PuV52/2008 | Peru | H12N5 | NAm | NAm | NAm (a) | NAm | NAm | NAm (a) |
| Charadriiformes | A/oystercatcher/Peru/MM152/2008 | Peru | H10N7 | NAm | NAm | N/D | NAm | SAm | NAm (a) |
| Charadriiformes | A/gull/Peru/CH02/2009 | Peru | H13N2 | EAs (Sh.) | NAm | NAm (a) | Sh. | EAs (Sh.) | Sh. (a) |
| Charadriiformes | A/curlew/Peru/PuV178/2009 | Peru | H3N1 | NAm | NAm | NAm (a) | NAm | NAm | NAm (a) |
| Charadriiformes | A/gull/Peru/PuV172/2009 | Peru | H1N1 | NAm | NAm | NAm (a) | NAm | NAm | NAm (a) |
| Charadriiformes | A/ruddy_turnstone/Peru/PuV181/2009 | Peru | H3N1 | NAm | NAm | NAm (a) | NAm | NAm | NAm (a) |
| Charadriiformes | A/ruddy_turnstone/Peru/PuV182/2009 | Peru | H3N1 | NAm | NAm | NAm (a) | NAm | NAm | NAm (a) |
| Charadriiformes | A/whimbrel/Peru/CH27/2009 | Peru | H1N1 | NAm | NAm | NAm (a) | NAm | NAm | NAm (a) |
| Charadriiformes | A/black_skimmer/Peru/CH55/2010 | Peru | H13N2 | EAs (Sh.) | NAm | Sh. | Sh. | EAs (Sh.) | Sh. (a) |
| Charadriiformes | A/gull/Peru/CH134/2010 | Peru | N6 | NAm | NAm | NAm (a) | NAm | NAm | NAm (a) |
| Charadriiformes | A/willet/Peru_CH49/2010 | Peru | H6N8 | NAm | NAm | NAm (a) | NAm | NAm | NAm (b) |
| Charadriiformes | A/gull/Peru/CH98/2010 | Peru | H13 | Sh. | NAm | SAm | Sh. | EAs (Sh.) | NAm (a) |
| Charadriiformes | A/gull/Peru/CH121/2010 | Peru | H13N2 | Sh. | NAm | NAm (a) | Sh. | EAs (Sh.) | Sh. (a) |
| Galliformes | A/chicken/Chile/184240_5/2002 | Chile | H7N3 | NAm | N/D | N/D | N/D | NAm | SAm (b) |
| Galliformes | A/chicken/Chile/4968/02 | Chile | H7N3 | N/D | N/D | N/D | N/D | SAm | SAm (b) |
| Galliformes | A/chicken/Chile/4977/02 | Chile | H7N3 | N/D | N/D | N/D | N/D | SAm | SAm (b) |
| Galliformes | A/turkey/Chile/4418/02 | Chile | H7N3 | N/D | N/D | N/D | N/D | SAm | SAm (b) |
| Galliformes | A/chicken/Chile/4322/02 | Chile | H7N3 | N/D | N/D | SAm | N/D | SAm | SAm (b) |
| Galliformes | A/chicken/Chile/184240_4322/2002 | Chile | H7N3 | N/D | SAm | SAm | SAm | SAm | SAm (b) |
| Galliformes | A/chicken/Chile/176822/02 | Chile | H7N3 | SAm | SAm | SAm | SAm | SAm | SAm (b) |
| Galliformes | A/chicken/Chile/184240_1/2002 | Chile | H7N3 | SAm | SAm | SAm | SAm | SAm | SAm (b) |
| Galliformes | A/chicken/Chile/4957/02 | Chile | H7N3 | SAm | SAm | SAm | SAm | SAm | SAm (b) |
| Galliformes | A/helmeted_guineafowl/Colombia/2440/2015 | Colombia | H11N2 | NAm | NAm | NAm (a) | NAm | NAm | NAm (a) |
| Gruiformes | A/red_fronted_coot/Chile/5/2013 | Chile | H3N6 | SAm | SAm | SAm | SAm | SAm | SAm (a) |
| Gruiformes | A/moorhen/Peru/120/2009 | Peru | H1N9 | NAm | NAm | NAm | NAm | NAm | NAm (a) |
| Pelecaniformes | A/pelican/Peru_MM24/2007 | Peru | H4N5 | NAm | NAm | N/D | NAm | NAm | NAm (b) |
| Pelecaniformes | A/egret/Peru_CH50/2010 | Peru | H6N8 | NAm | NAm | NAm (a) | NAm | NAm | NAm (b) |
| Sphenisciformes | A/Adelie_penguin/Antarctica/178/2013 | Antarctica | H11N2 | NAm | An. | An. | NAm | SAm | NAm (a) |
| Sphenisciformes | A/Adelie_penguin/Antarctica/184/2013 | Antarctica | H11N2 | NAm | An. | An. | NAm | SAm | NAm (a) |
| Sphenisciformes | A/Adelie_penguin/Antarctica/226/2013 | Antarctica | H11N2 | NAm | An. | An. | NAm | SAm | NAm (a) |
| Tinaniformes | A/red-winged_tinamou/Argentina/MP1/2008 | Argentina | H1N1 | SAm | SAm | SAm | SAm | SAm | SAm (b) |
