# Supplementary material for: Evidence of a fixed internal gene constellation in influenza A viruses isolated from wild birds in Argentina (2006–2016)
Source: Emerg Microbes Infect. 2018 Nov 28;7:194. doi: 10.1038/s41426-018-0190-2 (PMC6258671; doi:10.1038/s41426-018-0190-2)
Supplement: Supplementary file 7 — Supplementary Table 6 [file 41426_2018_190_MOESM7_ESM.doc]

STable 6. Sequence derived phenotype markers from Argentinean LPAIV isolated from 2006 to 2016.

|  | RESISTANCE^a^ | | | | | | |  | ENHANCED TRANSMISSION^b^ | | | | | |  | INCREASED VIRULENCE^c^ | | | |  | SEVERITY^d^ |
| --- | --- | --- | --- | --- | --- | --- | --- | --- | --- | --- | --- | --- | --- | --- | --- | --- | --- | --- | --- | --- | --- |
| Virus | M2 | | | | |  | NA |  | PA |  | PB2 | | | |  | PB1-F2 |  | PB2 | |  | NS1 |
|  | 26 | 27 | 30 | 31 | 34 |  | 274 |  | 409 |  | 199 | 661 | 667 | 702 |  | 66 |  | 627 | 701 |  | 92 |
| 432/H1N1* | L | V | A | S | G |  | **Y** |  | S |  | A | A | V | K |  | **S** |  | E | D |  | D |
| 32/H4N2* | L | V | A | S | G |  | H |  | S |  | A | A | V | K |  | **S** |  | E | D |  | D |
| 48/H4N6* | L | V | A | S | G |  | Q |  | S |  | A | A | V | K |  | **S** |  | E | D |  | D |
| 91/H4N6* | L | V | A | S | G |  | Q |  | S |  | A | A | V | K |  | **S** |  | E | D |  | D |
| 1227/H4N6* | L | V | A | S | G |  | Q |  | S |  | A | A | V | K |  | **S** |  | E | D |  | D |
| 25/H4N8 | L | V | A | S | G |  | I |  | S |  | A | A | V | K |  | **S** |  | E | D |  | D |
| 1737/H5N3* | L | V | A | S | G |  | I |  | S |  | A | A | V | K |  | **S** |  | E | D |  | D |
| 272/H6N2* | L | V | A | S | G |  | H |  | S |  | A | A | V | K |  | **S** |  | E | D |  | D |
| 557/H6N2 | L | V | A | S | G |  | H |  | S |  | A | A | V | K |  | **S** |  | E | D |  | D |
| 925/H6N2 | L | V | A | S | G |  | H |  | S |  | A | A | V | K |  | **S** |  | E | D |  | D |
| 1977/H6N2 | L | V | A | S | G |  | H |  | S |  | A | A | V | K |  | **S** |  | E | D |  | D |
| 49/H6N2* | L | V | A | S | G |  | H |  | S |  | A | A | V | K |  | **S** |  | E | D |  | D |
| 52/H6N2* | L | V | A | S | G |  | H |  | S |  | A | A | V | K |  | N |  | E | D |  | D |
| 1174A/H6N2* | L | V | A | S | G |  | I |  | S |  | A | A | V | K |  | **S** |  | E | D |  | D |
| 269/H6N8 | L | V | A | S | G |  | I |  | S |  | A | A | V | K |  | **S** |  | E | D |  | D |
| 575/H6N8 | L | V | A | S | G |  | I |  | S |  | A | A | V | K |  | **S** |  | E | D |  | D |
| 188/H7N7* | L | V | A | S | G |  | I |  | S |  | A | A | V | K |  | **S** |  | E | D |  | D |
| 1588/H7N9* | L | V | A | S | G |  | A |  | S |  | A | A | V | K |  | **S** |  | E | D |  | D |
| 559/H9N2 | L | V | A | S | G |  | H |  | S |  | A | A | V | K |  | **S** |  | E | D |  | D |
| 171/H10N7* | L | V | A | S | G |  | I |  | S |  | A | A | V | K |  | **S** |  | E | D |  | D |
| 175/H10N7* | L | V | A | S | G |  | I |  | S |  | A | A | V | K |  | **S** |  | E | D |  | D |
| LDC4/H13N9 | L | V | A | S | G |  | A |  | S |  | A | A | V | K |  | **S** |  | E | D |  | D |
| NAm lineage | L (200 out 200, 100%) | V (200 out 200, 100%) | A (200 out 200, 100%) | S (200 out 200, 100%) | G (200 out 200, 100%) |  | # |  | S (88 out 95, 92,6%) |  | A (113 out 113, 100%) | A (109 out 113, 96,4%) | V (104 out 113, 92%) | K (110 out 113, 97,3%) |  | **S (45 out 78, 57,7%)** |  | E (112 out 113, 99%) | D (113 out 113, 100%) |  | D (111 out 111, 100%) |
| EAs lineage | L (95 out 95, 100%) | V (95 out 95, 100%) | A (95 out 95, 100%) | S (95 out 95, 100%) | G (95 out 95, 100%) |  | # |  | S (54 out 61, 88,5%) |  | A (96 out 97, 99%) | A (88 out 97, 90,7%) | V (91 out 97, 93,8%) | K (93 out 97, 95,9%) |  | N (63 out 77, 81,8%) |  | E (96 out 97, 99%) | D (96 out 97, 99%) |  | D (96 out 99, 96,9%) |
|  |  |  |  |  |  |  |  |  |  |  |  |  |  |  |  |  |  |  |  |  |  |
| * New fifteen IAVs isolates from Argentina from this study are in bold | | | | | |  |  |  |  |  |  |  |  |  |  |  |  |  |  |  |  |
| Mutations marked in bold are associated with adamantane/olseltavimir resistance, enhanced transmission, increased virulence or severity | | | | | | | | | | | | | | | | |  |  |  |  |  |
| ^a^ Adamantane Resistance (M2: L26F, V27A, A30T, S31N and G34E) and Olseltamivir Resistance (NA: H274Y) | | | | | | | | | | | | | | | | | | | | | |
| ^b^ Enhanced Transmission (PA: S409N; PB2: A199S, A661T, V667I and K702R) | | | | | | | | | |  |  |  |  |  |  |  |  |  |  |  |  |
| ^c^ Increased Virulence (PB1-F2: N66S; PB2: E627K and D701N) | | | | | |  |  |  |  |  |  |  |  |  |  |  |  |  |  |  |  |
| ^d^ Severity (NS1: T92E) | | | | | | | | | | | | |  |  |  |  |  |  |  |  |  |
| # NAm and EAs lineages have the same aminoacid at position 274 as SAm lineage for N2 (H), N6 (Q) and N9 (A). N1: Y in 161 out 161 (100%) of NAm sequences and Y in 160 out 165 (97%) of EAs sequences. N3: I in 236 out 237 (99,6%) of NAm sequences and I in 102 out 102 (100%) of EAs sequences. N7: I in 121 out 122 (99,1%) of NAm sequences and I in 78 out 78 (100%) of EAs sequences. N8: I in 144 out 146 (98,6%) of NAm sequences and I in 45 out 47 (95,7%) of EAs sequences. | | | | | | | | | | | | | | | | | | | | | |
