# Supplementary material for: Evidence of a fixed internal gene constellation in influenza A viruses isolated from wild birds in Argentina (2006–2016)
Source: Emerg Microbes Infect. 2018 Nov 28;7:194. doi: 10.1038/s41426-018-0190-2 (PMC6258671; doi:10.1038/s41426-018-0190-2)
Supplement: Supplementary file 8 — Supplementary Figure 1 [file 41426_2018_190_MOESM8_ESM.pdf]

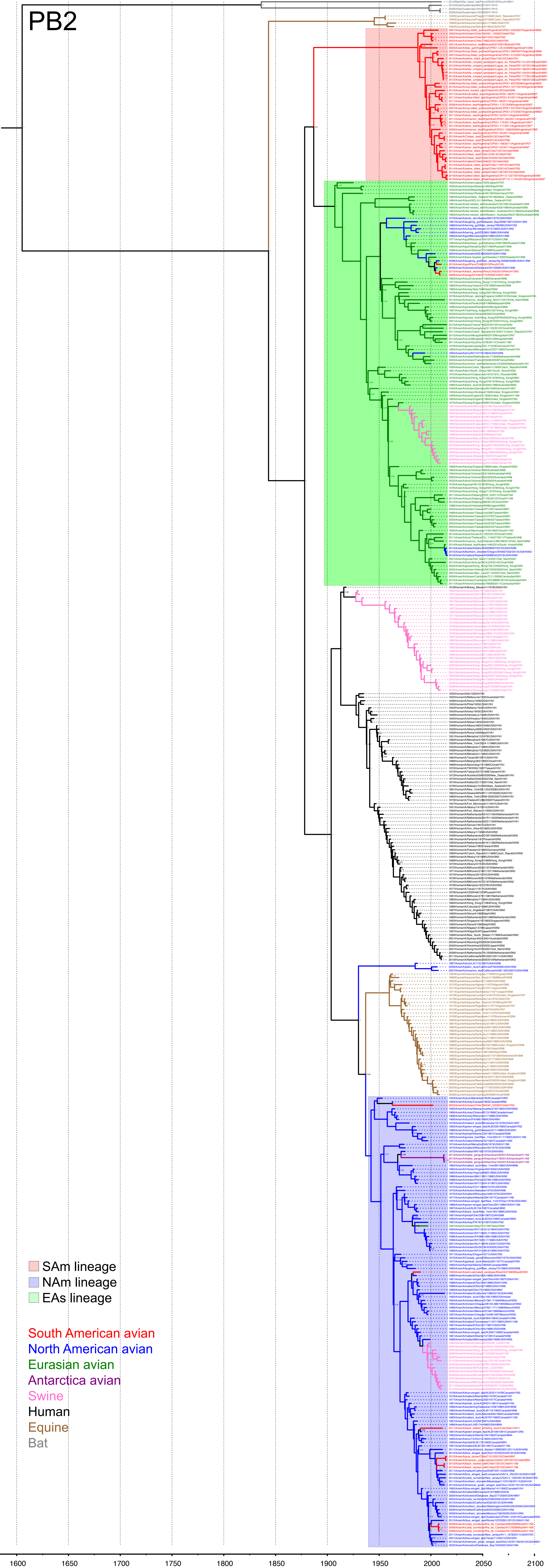
PB2
SAm lineage
NAm lineage
EAs lineage
South American avian
North American avian
Eurasian avian
Antarctica avian
Swine
Human
Equine
Bat
1600
1650
1700
1750
1800
1850
1900
1950
2000
2050
2100
