# Supplementary figures and images for: Evidence of a fixed internal gene constellation in influenza A viruses isolated from wild birds in Argentina (2006–2016)
Source: Emerg Microbes Infect. 2018 Nov 28;7:194. doi: 10.1038/s41426-018-0190-2 (PMC6258671; doi:10.1038/s41426-018-0190-2)

PB1

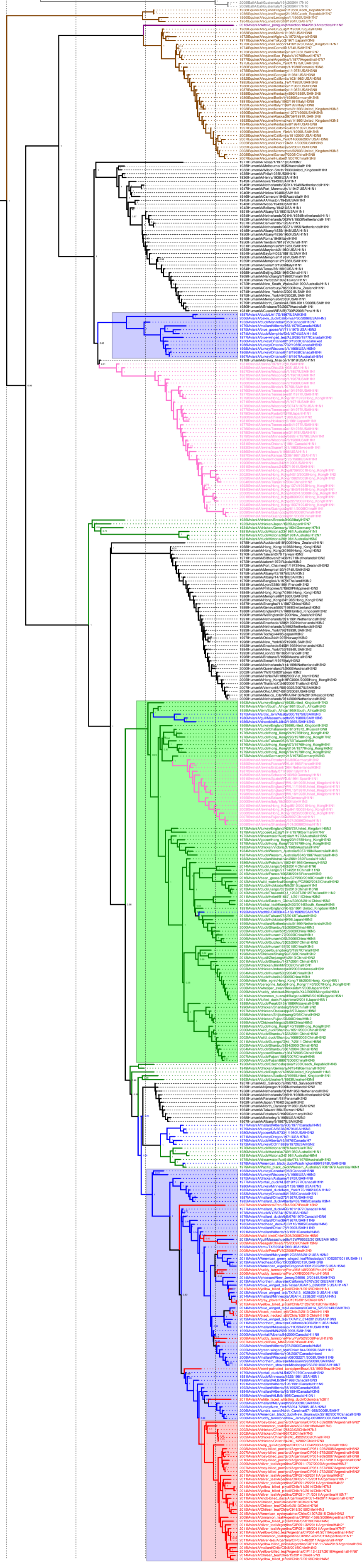

1750

1800

1850

1900

1950

2000

2050

Supplement: Supplementary file 9 — Supplementary Figure 2 [file 41426_2018_190_MOESM9_ESM.pdf]

PA

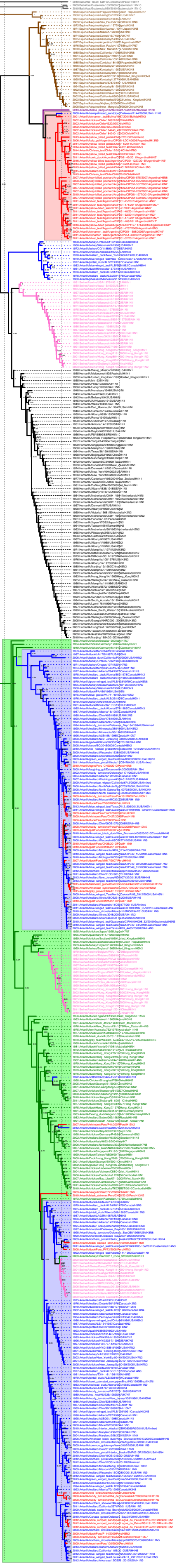

1700

1800

1900

2000

2100

Supplement: Supplementary file 10 — Supplementary Figure 3 [file 41426_2018_190_MOESM10_ESM.pdf]

•

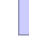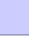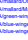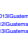

Supplement: Supplementary file 11 — Supplementary Figure 4 [file 41426_2018_190_MOESM11_ESM.pdf]

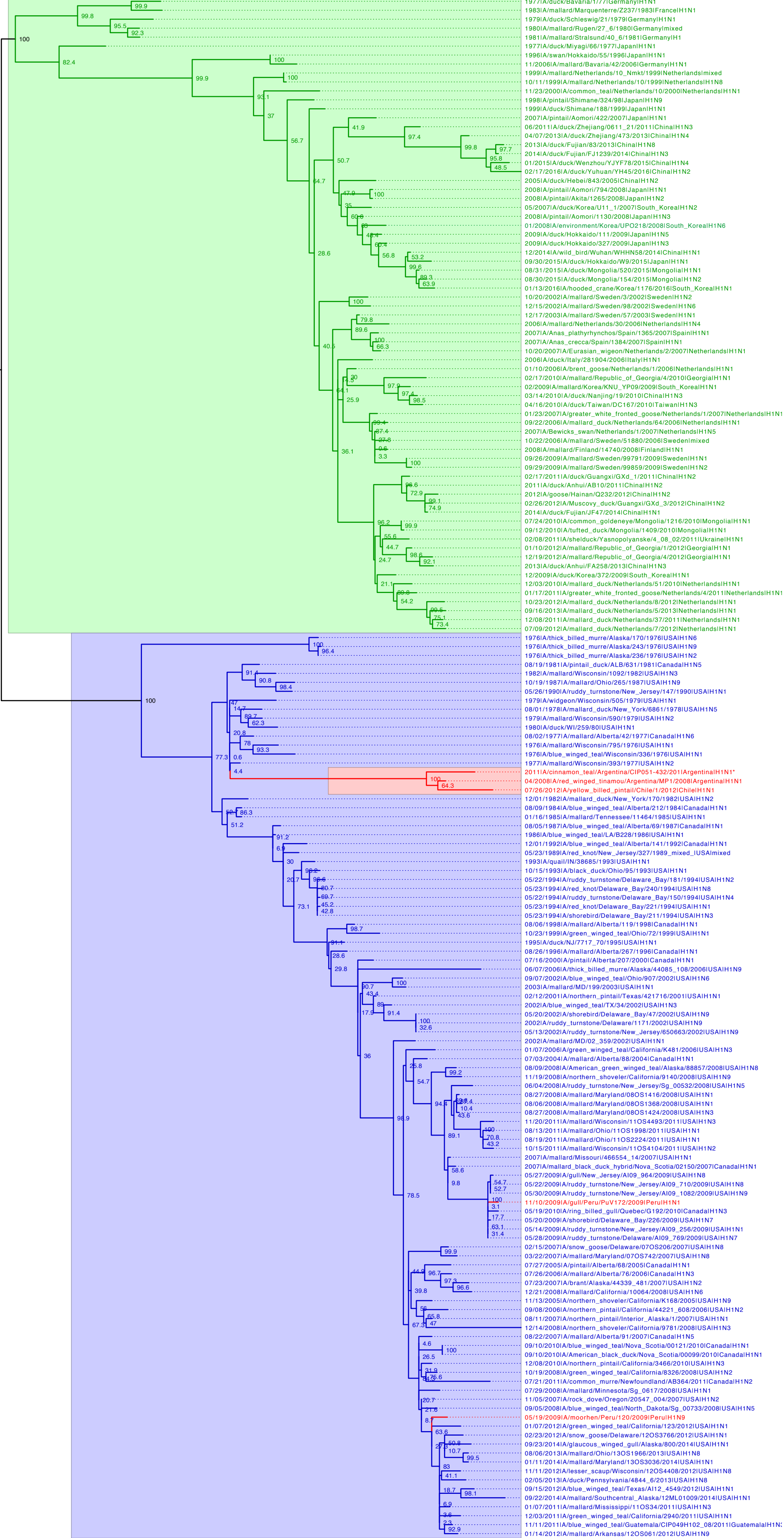

Supplement: Supplementary file 14 — Supplementary Figure 7 [file 41426_2018_190_MOESM14_ESM.pdf]

H5

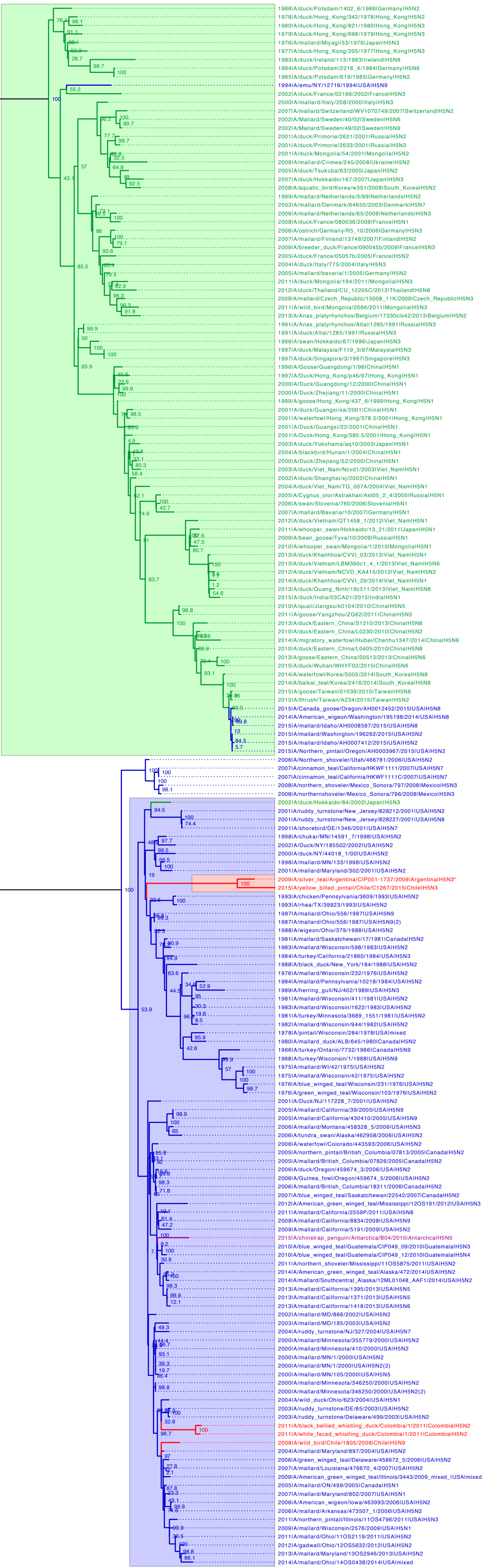

0.03

Supplement: Supplementary file 15 — Supplementary Figure 8 [file 41426_2018_190_MOESM15_ESM.pdf]

H7

0.05

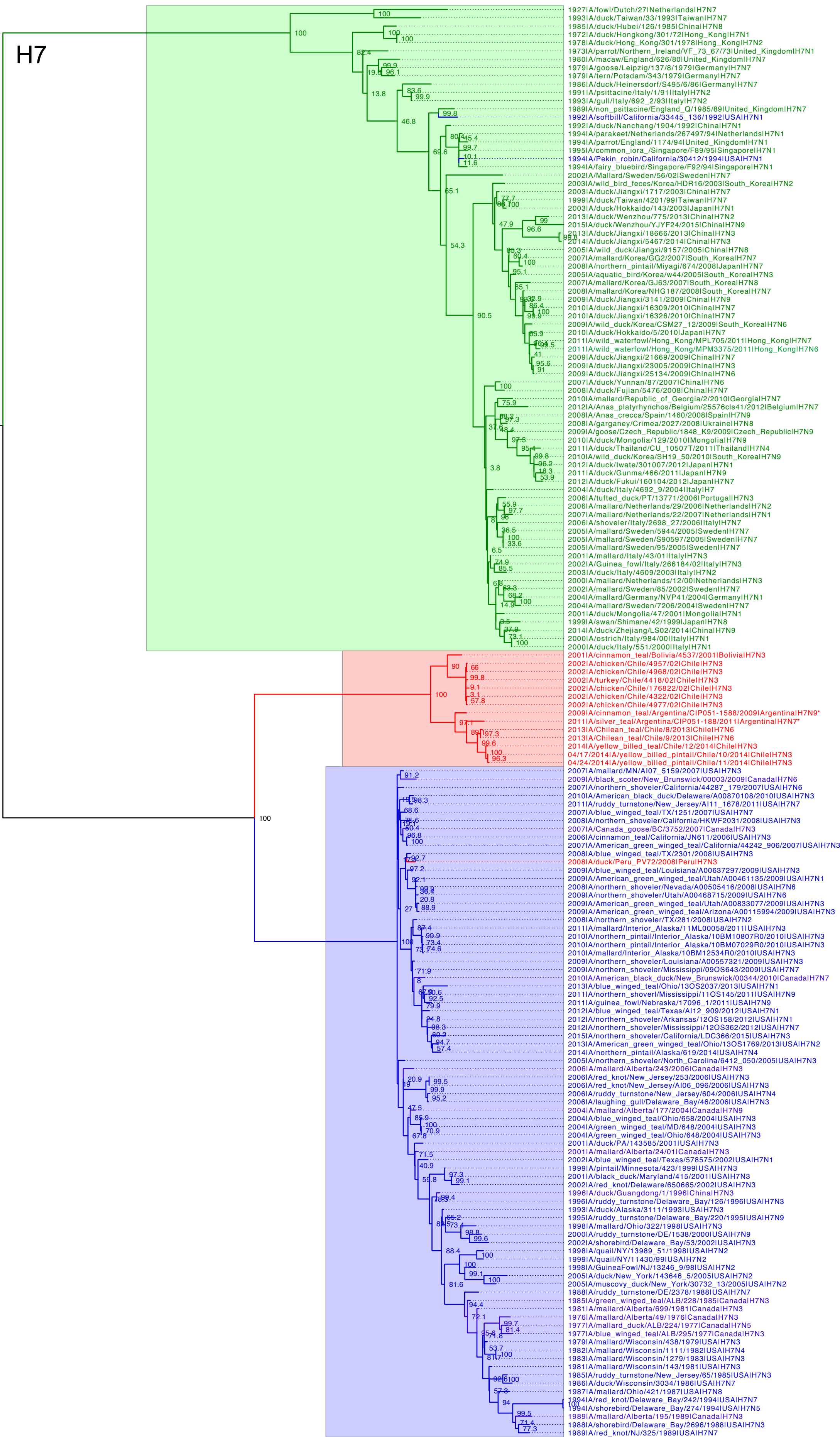

Supplement: Supplementary file 16 — Supplementary Figure 9 [file 41426_2018_190_MOESM16_ESM.pdf]

H10

1

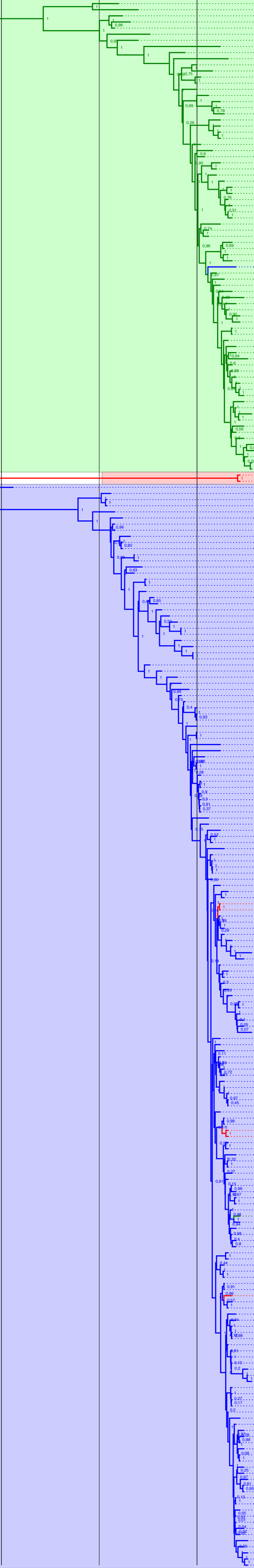

# 2050

Supplement: Supplementary file 17 — Supplementary Figure 10 [file 41426_2018_190_MOESM17_ESM.pdf]

H4

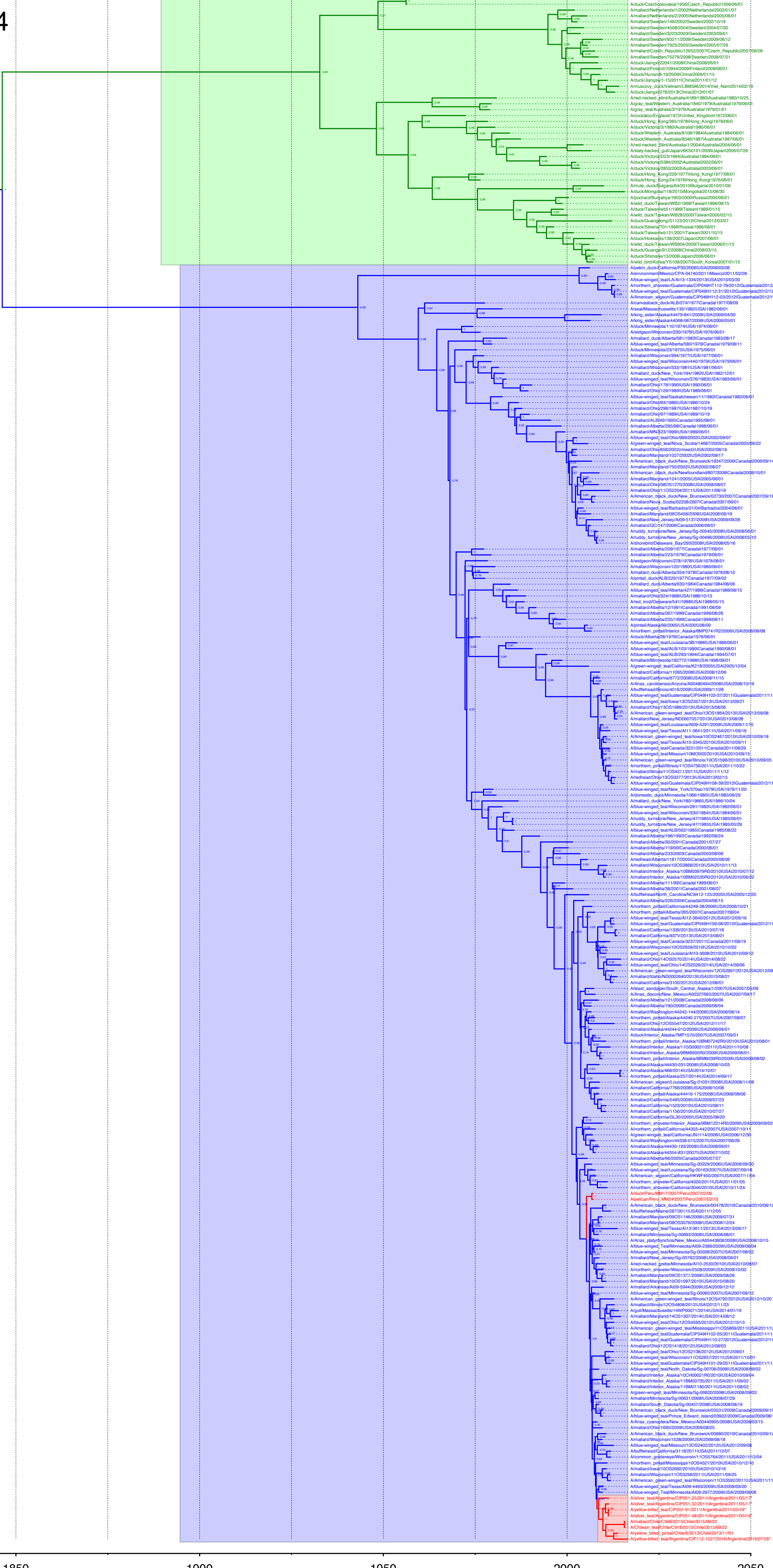

Supplement: Supplementary file 18 — Supplementary Figure 11 [file 41426_2018_190_MOESM18_ESM.pdf]

H6

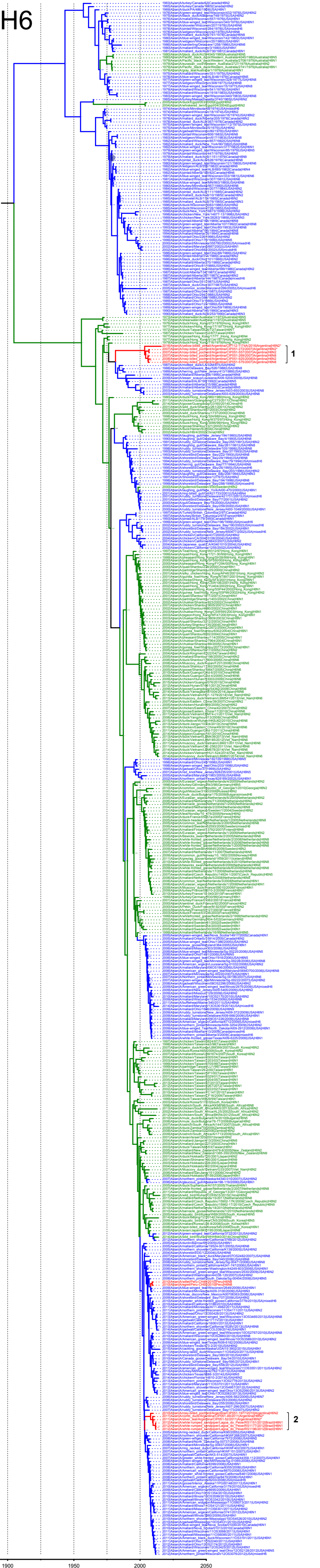

Supplement: Supplementary file 19 — Supplementary Figure 12 [file 41426_2018_190_MOESM19_ESM.pdf]

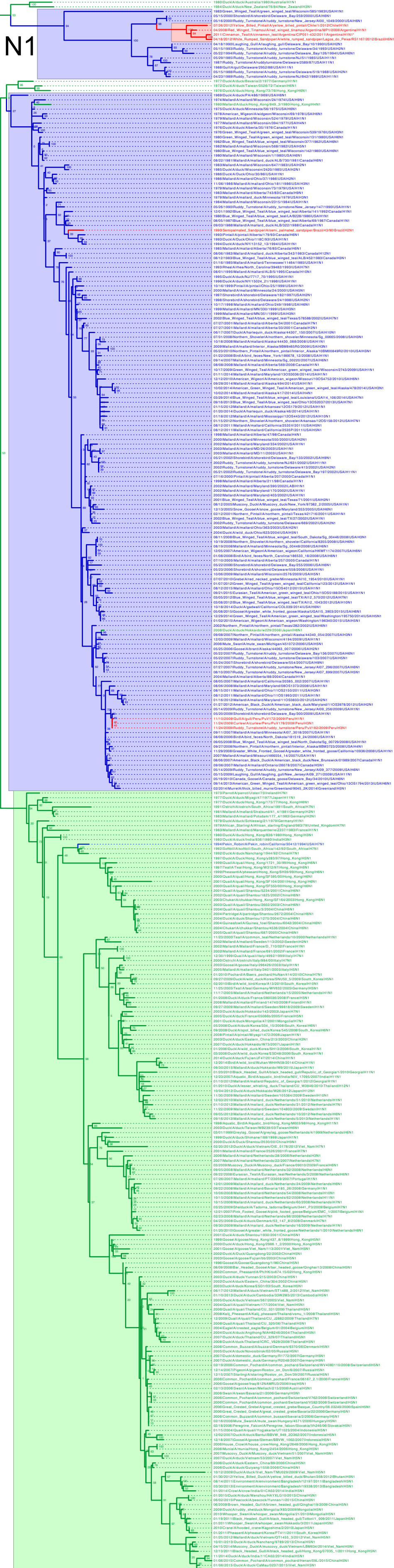

Supplement: Supplementary file 20 — Supplementary Figure 13 [file 41426_2018_190_MOESM20_ESM.pdf]

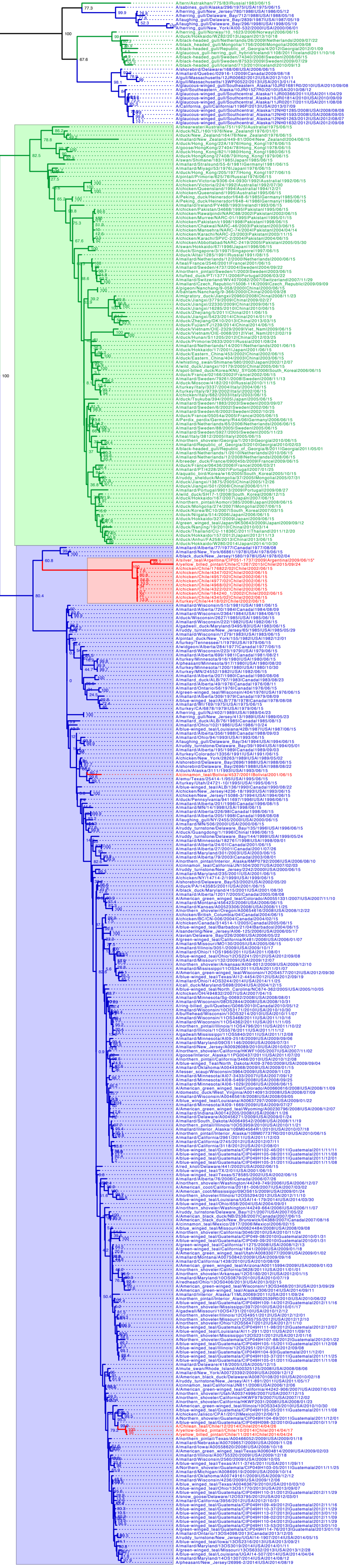

Supplement: Supplementary file 22 — Supplementary Figure 15 [file 41426_2018_190_MOESM22_ESM.pdf]

N6

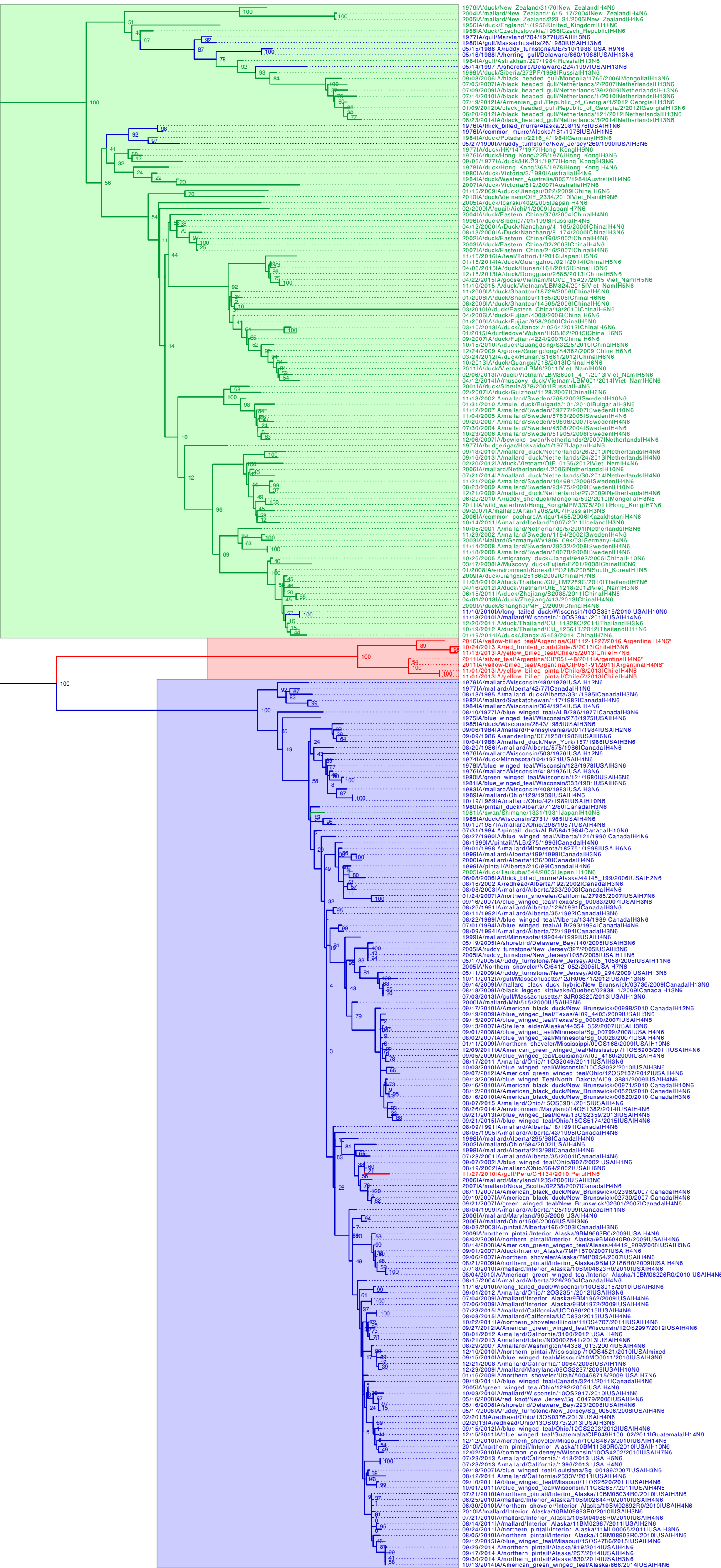

Supplement: Supplementary file 23 — Supplementary Figure 16 [file 41426_2018_190_MOESM23_ESM.pdf]

N9

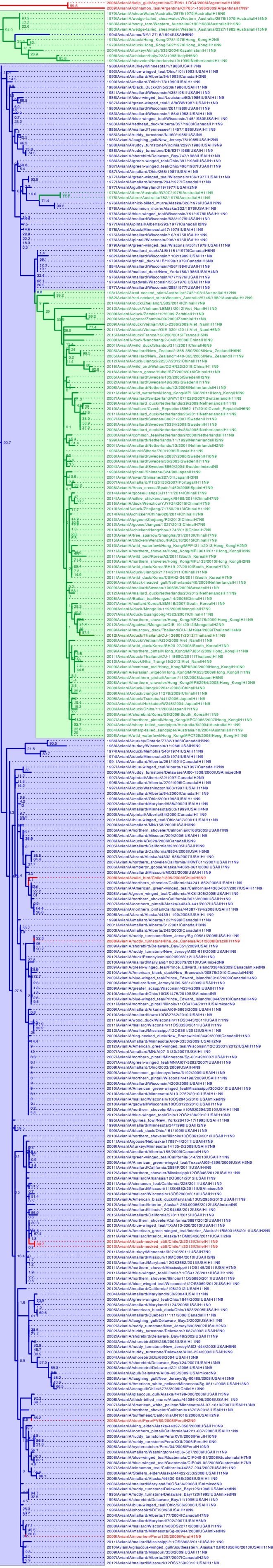

0.03

Supplement: Supplementary file 24 — Supplementary Figure 17 [file 41426_2018_190_MOESM24_ESM.pdf]

N7

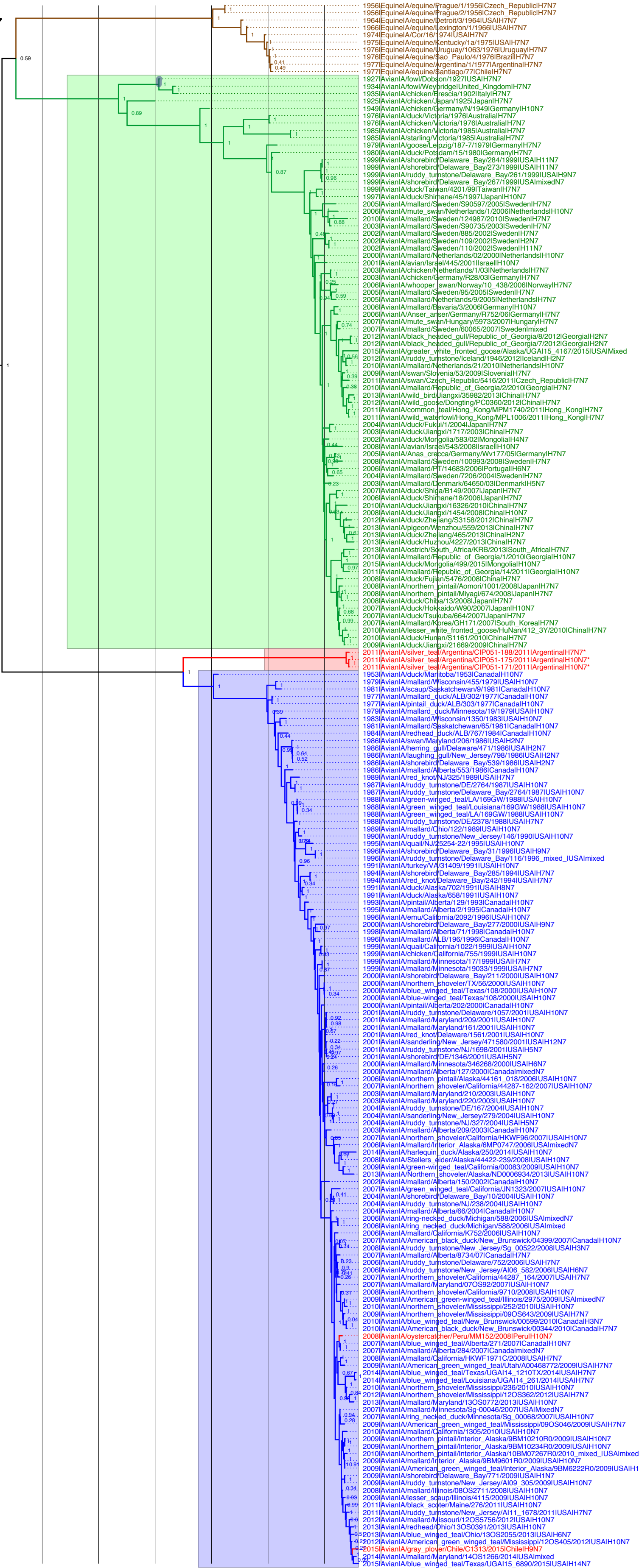

1850 1900 1950 2000 2050

Supplement: Supplementary file 25 — Supplementary Figure 18 [file 41426_2018_190_MOESM25_ESM.pdf]
