# Supplementary material for: Evidence of a fixed internal gene constellation in influenza A viruses isolated from wild birds in Argentina (2006–2016)
Source: Emerg Microbes Infect. 2018 Nov 28;7:194. doi: 10.1038/s41426-018-0190-2 (PMC6258671; doi:10.1038/s41426-018-0190-2)
Supplement: Supplementary file 13 — Supplementary Figure 6 [file 41426_2018_190_MOESM13_ESM.pdf]

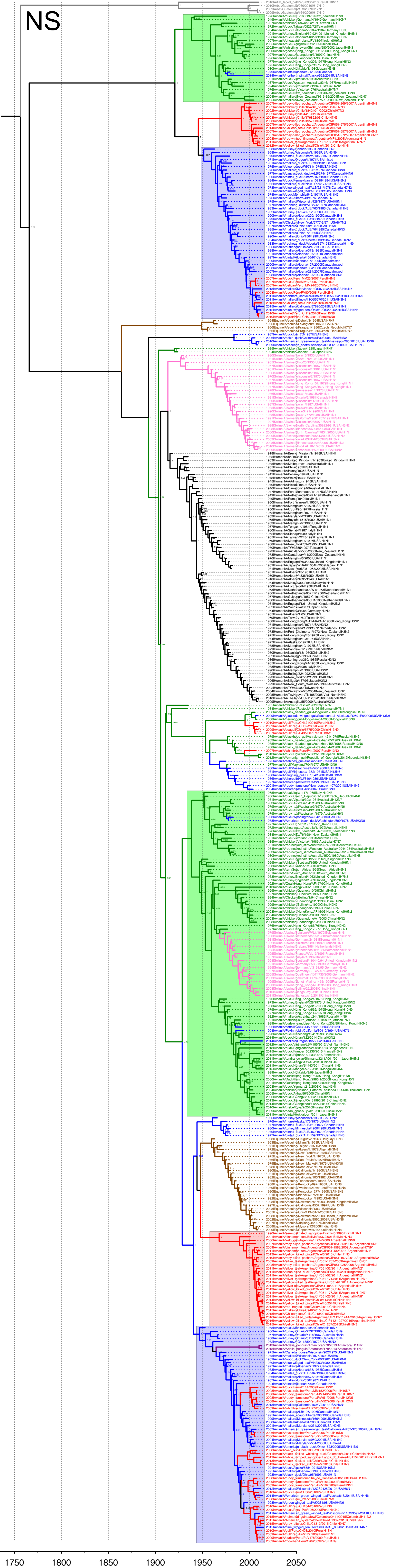
NS
1750
1800
1850
1900
1950
2000
2050
