# Supplementary material for: Evidence of a fixed internal gene constellation in influenza A viruses isolated from wild birds in Argentina (2006–2016)
Source: Emerg Microbes Infect. 2018 Nov 28;7:194. doi: 10.1038/s41426-018-0190-2 (PMC6258671; doi:10.1038/s41426-018-0190-2)
Supplement: Supplementary file 28 — Supplementary Figure 21 [file 41426_2018_190_MOESM28_ESM.pdf]

```
                  10        20        30        40        50        60        70        80        90
             ....|....|....|....|....|....|....|....|....|....|....|....|....|....|....|....|....|....|
432/H1N1*    MEDFVRQCFNPMIVELAEKAMKEYGEDPKIETNKFAAICTHLEVCFMYSDFHFIDERGESVIVESGDPNALLKHRFEIIEGRDRTMAWTV
32/H4N2*     ..........................................................................................
48/H4N6*     ............................................................I.............................
91/H4N6*     ............................................................I.............................
1227/H4N6*   .G..........................................................I.............................
25/H4N8*     ............................................................I.............................
1737/H5N3*   ...........I..............................................................................
272/H6N2*    ..........................................................................................
557/H6N2     ..........................................................................................
925/H6N2     ..................R.......................................................................
1977/H6N2    ............................................................I.............................
49/H6N2*     ............................................................I.............................
52/H6N2*     ..........................................................................................
1174A/H6N2*  .G............................................................I...........................
269/H6N8     .....................................................................................I....
575/H6N8     ..........................................................................................
188/H7N7*    .............................................L............................................
1588/H7N9*   ...........I..............................................................................
559/H9N2     .........................................................S................................
171/H10N7*   ............................................................I.............................
175/H10N7*   .............................................L............................................
LDC4/H13N9   .G....................................................N.....I.............................

                 100       110       120       130       140       150       160       170       180
             ....|....|....|....|....|....|....|....|....|....|....|....|....|....|....|....|....|....|
432/H1N1*    VNSICNTTGVEKPRFLPDLYDYKENRFIEIGVTRREVHIYYLEKANKIKSEKTHIHIFSFTGEEMATKADYTLDEESRARIKTRLFTIRQ
32/H4N2*     .............K............................................................................
48/H4N6*     .............K............................................................................
91/H4N6*     .............K............................................................................
1227/H4N6*   .............K............................................................................
25/H4N8*     .............K............................................................................
1737/H5N3*   .............K............................................................................
272/H6N2*    .............K.............V..............................................................
557/H6N2     .............K.............V..............................................................
925/H6N2     .............K............................................................................
1977/H6N2    .............K............................................................................
49/H6N2*     .............K............................................................................
52/H6N2*     ..........................................................................................
1174A/H6N2*  .............K............................................................................
269/H6N8     .............K............................................................................
575/H6N8     ..........................................................................................
188/H7N7*    .............K............................................................................
1588/H7N9*   .............K............................................................................
559/H9N2     .............K....................................................S.......................
171/H10N7*   .............K............................................................................
175/H10N7*   .............K............................................................................
LDC4/H13N9   .............K............................................................................

                 190       200       210       220       230       240       250
             ....|....|....|....|....|....|....|....|....|....|....|....|....|....|...
432/H1N1*    EMASRGLWDSFVSPREAKRQLKKDLKSQEQCVGLPNKVSHRTSPALKTLEPMWMDSNRTAALRASFLKCPKK*
32/H4N2*     ...............................A...T...........................E........*
48/H4N6*     .................R.............A...T.....................................*
91/H4N6*     .................R.............A...T.....................................*
1227/H4N6*   ...............................A...T.....................................*
25/H4N8*     .................R.............A...T.....................................*
1737/H5N3*   ...............................A...T.....................................*
272/H6N2*    ...............................A...T.....................................*
557/H6N2     ...............................A...T.....................................*
925/H6N2     ...............................A...T.....................................*
1977/H6N2    ....K..........................A..LT.....................................*
49/H6N2*     ...............................A...T.....................................*
52/H6N2*     ...............................A...T.....................................*
1174A/H6N2*  ............................G..A...T...................................R.*
269/H6N8     ...............................A...T.....................................*
575/H6N8     ...................................T...............................R.....*
188/H7N7*    ...............................A...T.....................................*
1588/H7N9*   ...............................A...T.....................................*
559/H9N2     .....S........K..R.............AS..T.....................................*
171/H10N7*   .................R.............A...T.....................................*
175/H10N7*   ...............................A...T.....................................*
LDC4/H13N9   ...............................A...T.....................................*
```
